# CRISPR-Cas12a has both *cis*- and *trans*-cleavage activities on single-stranded DNA

**DOI:** 10.1038/s41422-018-0022-x

**Published:** 2018-03-12

**Authors:** Shi-Yuan Li, Qiu-Xiang Cheng, Jia-Kun Liu, Xiao-Qun Nie, Guo-Ping Zhao, Jin Wang

**Affiliations:** 1 Key Laboratory of Synthetic Biology, Institute of Plant Physiology and Ecology, Shanghai Institutes for Biological Sciences, Chinese Academy of Sciences, 200032 Shanghai, China; 2 University of Chinese Academy of Sciences, 100049 Beijing, China; 3 Shanghai Tolo Biotechnology Company Limited, 200233 Shanghai, China; 4 Department of Microbiology and Li KaShing Institute of Health Sciences, The Chinese University of Hong Kong, Prince of Wales Hospital, Shatin, New Territories, Shatin, New Territories, China

**Keywords:** Molecular biology, Biological techniques

Dear Editor,

The CRISPR-associated protein Cas12a (previously known as Cpf1), which is an endonuclease from the type V-A CRISPR system, has been applied in both in vivo genome editing and in vitro DNA assembly.^1–3^ Cas12a is guided by a single CRISPR RNA (crRNA) with a T-rich protospacer adjacent motif (PAM) sequence to cleave double-stranded DNA (dsDNA) targets, generating sticky ends. Different from Cas9, Cas12a cleaves both the target and non-target strands of a targeted dsDNA by a single active site in the RuvC catalytic pocket^4–6^ (Supplementary information, Figure S12a). Besides, Cas12a also processes precursor crRNAs to generate mature crRNAs.^7^ However, the cleavage activity of Cas12a on single-stranded DNA (ssDNA) targets is less understood.

To investigate the ssDNA cleavage feature of Cas12a, we employed FnCas12a to cleave short ssDNAs that were labelled with 5(6)-carboxyfluorescein (FAM) on the 3′ terminus and found that the ssDNA cleavage sites were near the 22nd base (i.e., from the 21st to the 23rd), counting from the first 3′-base that was paired with the crRNA guide sequence (Supplementary information, Figure S1a and b and Tables S2 and 3). The cleavage did not require the existence of a PAM sequence in the targeted ssDNA (Supplementary information, Figure S1a and b). In addition, the same cleavage sites were obtained with crRNAs having guide sequences as short as 10 nucleotides (nt) (Supplementary information, Figure S1c and d), which indicates that Cas12a could cleave ssDNA at sites outside of the recognition sequence. We then tested Cas12a cleavage efficiency on ssDNA and dsDNA substrates, and cleavage of ssDNA was slower than that of dsDNA (Supplementary information, Figure S1e and f), whose PAM sequence may account for the higher efficiency.

We also performed the Cas12a cleavage experiment with a ssDNA target labelled at its 5′ terminus (target-DNMT1-3-R-FAM-5′). Surprisingly, no cleaved bands were observed at the predicted size (20 nt), but short (<6 nt) FAM-labelled products were generated (Fig. 1a). After careful analyses of experimental conditions, we found that only the ternary complex of Cas12a/crRNA/targeted ssDNA (or targeted dsDNA) was able to cleave the 5′-labelled target ssDNA (target-DNMT1-3), generating short FAM-labelled products (Fig. 1b and Supplementary information, Figure S2). The ternary complex also promiscuously cleaved collateral ssDNAs that had no complementarity to the crRNA guide sequence in the reaction system, generating short products (Fig. 1c and Supplementary information, Figure S3). As it is difficult to distinguish the precise length of the short *trans*-cleavage products via polyacrylamide gel electrophoresis, the FAM-labelled short products were purified and analysed by liquid chromatography-mass spectrometry. The results showed that 5′-FAM-labelled substrates were mainly *trans*-cleaved to 4 nt, while 2-nt products were observed for 3′-FAM-labelled substrates (Supplementary information, Figure S4).

**Fig. 1:**
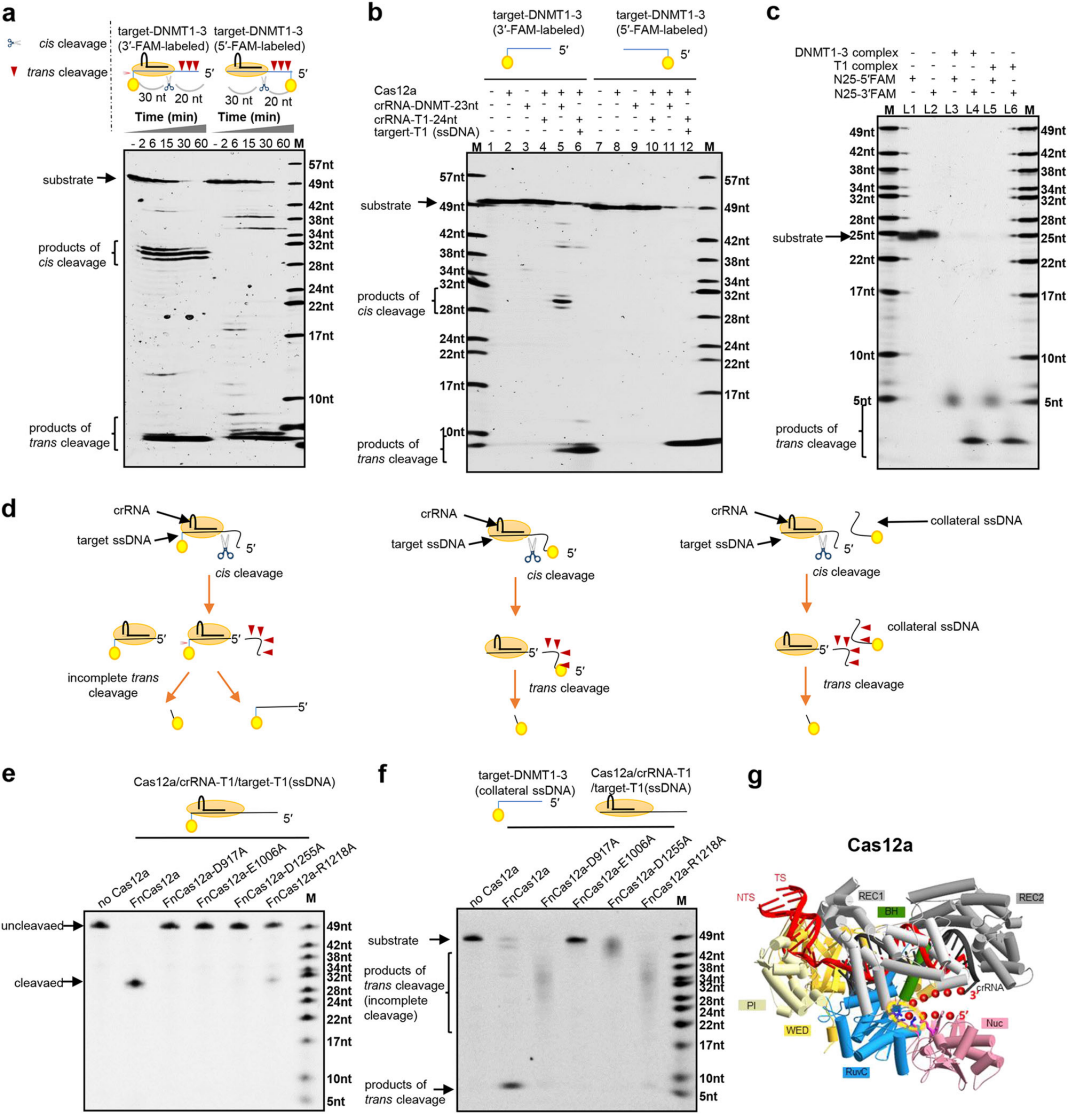
Determination of the ssDNA cleavage activities of the complex of Cas12a/crRNA/target DNA. **a** Time-course experiment of ssDNA (target-DNMT1-3) cleavage with crRNA-DNMT-23nt. The 3′-end FAM-labelled ssDNA was cleaved at expected sites (left); however, cleavage of 5′-FAM-labelled ssDNA generated no expected products (20 nt) but short oligonucleotides (<6 nt). **b** With a target-specific crRNA of crRNA-DNMT-23nt, Cas12a cleaved the 3′-FAM-labelled target-DNMT1-3 (ssDNA) at expected sites but cleaved the 5′-FAM-labelled target-DNMT1-3 (ssDNA) to short oligonucleotides. Collateral single-stranded target-DNMT1-3 (either 5′- or 3′-FAM-labelled) was cleaved to short oligonucleotides upon the formation of the ternary complex of Cas12a/crRNA-T1-24nt/target-T1 DNA. (**c**) Cleavage of random collateral ssDNA by the ternary complex of Cas12a/crRNA/target DNA. Random short single-stranded oligonucleotides (25 nt) were labelled with FAM at either the 5′-end or the 3′-end, obtaining N25-5′FAM and N25-3′FAM, respectively. The labelled random oligonucleotides were cleaved by the complex of Cas12a/crRNA-DNMT-23nt/target-DNMT1-3 (ssDNA) in lanes 3 and 4 or by the complex of Cas12a/crRNA-T1-24nt/target-T1 (ssDNA) in lanes 5 and 6, respectively. Lanes 1 and 2 showed synthesized random oligonucleotides that were labelled at the 5′-end and 3′-end, respectively. **d** Illustration of both *cis*- and *trans*-cleavage by the Cas12a complex in **a**–**c**. **e** *Cis*-cleavage of ssDNA (target-T1-R-FAM) by FnCas12a and its mutants. Mutations in D917, E1006, D1255 and R1218, four residues that are associated with the DNase activity, were generated. All tested mutants showed either completely lost or largely decreased *cis*-cleavage activity on target ssDNA. **f** *Trans*-cleavage of the 3′-FAM-labelled collateral ssDNA (target-DNMT1-3-R) by the ternary complexes of FnCas12a or its mutants (D917A, E1006A, D1255A and R1218A in FnCas12a). The *trans*-cleavage activity on collateral ssDNA was completely lost in E1006A and decreased in other mutants. **g** The ternary complex of Cas12a (PDB: 5B43) with proposed collateral ssDNA. Red dots represented the proposed positions of collateral ssDNA. Molecular graphic images were prepared using CueMol (http://www.cuemol.org). DNA was coloured in red, RNA was coloured in black and the RuvC catalytic pocket was indicated by dashed yellow circles

We called the promiscuous cleavage of collateral ssDNAs *trans*-cleavage to distinguish it from the programmable on-target cleavage of target ssDNA (namely, *cis*-cleavage), and the proposed ssDNA cleavage processes were illustrated in Fig. 1d. When the ssDNA substrate was labelled at the 5′ terminus, the *cis*-cleaved 5′-labelled ssDNA products became collateral ssDNAs in the reaction system and were subsequently *trans*-cleaved into short products, explaining the observed cleavage pattern for 5′-labelled ssDNA substrate. We observed the *trans*-cleavage products in addition to the *cis*-products for short 3′-labelled targeted ssDNAs (Fig. 1a). The majority of the ternary complex most likely remained bound to the targeted ssDNAs after *cis*-cleavage, protecting the labelled 3′-terminus from exposing the *trans*-activity sites of the Cas12a ternary complex.

Next, we tested nine randomly selected Cas12a proteins from different species in addition to the above tested FnCas12a (Supplementary information, Figures S5, 6a and Tables S1, 4 and 5), and all Cas12a proteins exhibited endonuclease activity on plasmid dsDNA (Supplementary information, Figure S6b), *cis*- (Supplementary information, Figure S6c) and *trans*-cleavage activities on ssDNA (Supplementary information, Figure S6d). This indicates that the *cis-* and *trans*-cleavage activities on ssDNA might be ubiquitous among Cas12a proteins.

When shortened targeted ssDNAs were tested, complexes with 18-nt target ssDNAs that lacked a cleavage site also showed *trans*-cleavage activity (Supplementary information, Figure S7a), indicating that *cis*-cleavage was not a prerequisite for *trans*-cleavage activity. *Trans*-cleavage was implemented by the endonuclease activity of the complex, as circular ssDNA (M13mp18) could also be *trans*-cleaved (Supplementary information, Figure S7b). Moreover, we found that all tested Cas12a complexes except the AsCas12a complex had *trans*-cleavage activity on collateral dsDNAs (Figure S8), and the activity of the LbCas12a, BoCas12a and Lb4Cas12a complexes was much higher.

To identify key residues involved in ssDNA cleavage of both targeted and collateral ssDNAs, we mutated several candidate residues in FnCas12a to alanines, including those related to the RNase activity (H843, K852 and K869)^7^ and those responsible for dsDNA cleavage (D917, E1006, D1255 and R1218) (refs. ^1,7–9^ and Supplementary information, Figure S9). Both *cis*- and *trans*-cleavage of ssDNA were unaffected in the RNase activity-related mutants (Supplementary information, Figure S10), but the activities were completely lost or remarkably decreased with mutations in either the RuvC domain (D917A, E1006A and D1255A mutations in FnCas12a) or the Nuc domain (R1218A mutation in FnCas12a) (Fig. 1e, f). Recent studies showed that the RuvC catalytic pocket of both C2c1 and Cas12a was responsible for the cleavage of both strands of targeted dsDNA,^4–6^ leading us to propose that targeted ssDNAs were *cis*-cleaved by this catalytic pocket (Supplementary information, Figures S11a, b, d, e and S12c). Moreover, according to the structure of the C2c1-crRNA-excess DNA complex (Supplementary information, Figure S11c),^4^ *trans*-cleavage of collateral ssDNAs could also be achieved by the same catalytic pocket (Fig. 1g, and Supplementary information, Figure S12b and d) in the Cas12a ternary complex. The RuvC catalytic pocket is consistently exposed in the bilobed structure of the ternary Cas12a complexes^8^ (Supplementary information, Figure S11f) but not in the structurally dynamic Cas12a monomer^9^ nor in the triangle-shaped binary structure^9^ (Supplementary information, Figure S11g), allowing for *trans*-cleavage of collateral ssDNAs.

Taken together, we here show that the cleavage activities on ssDNAs, including both *cis*- and *trans*-cleavage, are ubiquitous among Cas12a proteins, and proposed cleavage models are shown in Supplementary information, Figure S12. Notably, Cas12a is so far the first characterized Cas protein whose ternary complex has *trans*-ssDNA cleavage activity. Considering the fact that a large number of single-stranded viruses exist in the environment, Cas12a may get the ssDNA cleavage activity during evolution and may function as a powerful tool to prevent the invasion of foreign ssDNAs. In addition to Cas12a, Cas13a (previously known as C2c2), an RNA-guided and RNA-targeting CRISPR effector from the class 2 type VI CRISPR system, was found with the *trans*-cleavage activity on RNA.^10–12^ Recently, this characteristic of Cas13a was successfully employed for rapid and sensitive nucleic acid detection.^13^ Therefore, the *trans*-cleavage activity of Cas12a characterized in this study may also be utilized in potential biotechnological applications in a similar manner. Since different Cas12a complexes show various *trans*-cleavage activity on dsDNA, the in-depth mechanism needs further investigation.

### Electronic supplementary material

Supplementary information

## Electronic supplementary material

**Supplementary information** accompanies for this paper at 10.1038/s41422-018-0022-x.
