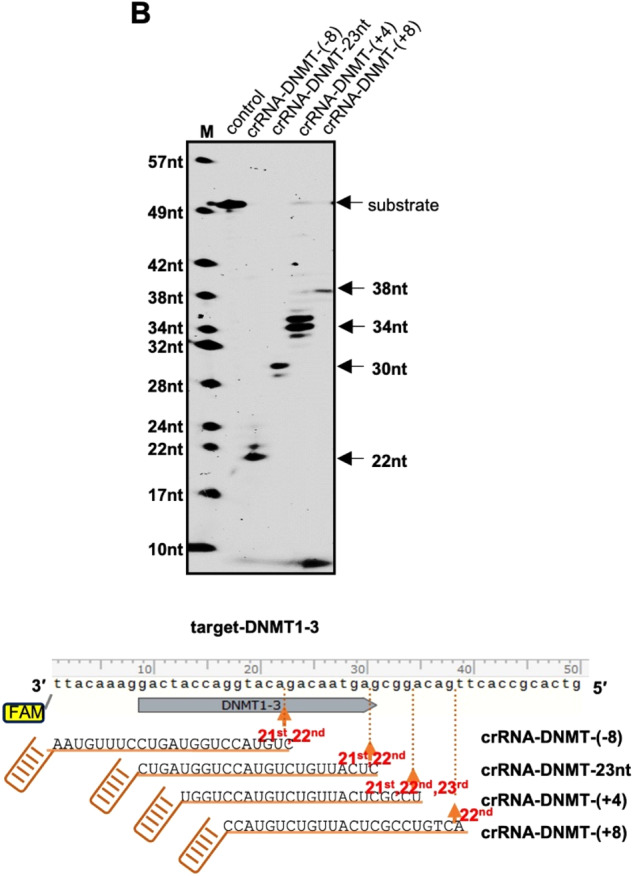# Author Correction: CRISPR-Cas12a has both *cis*- and *trans*-cleavage activities on single-stranded DNA

**DOI:** 10.1038/s41422-024-00927-2

**Published:** 2024-01-23

**Authors:** Shi-Yuan Li, Qiu-Xiang Cheng, Jia-Kun Liu, Xiao-Qun Nie, Guo-Ping Zhao, Jin Wang

**Affiliations:** 1 Key Laboratory of Synthetic Biology, Institute of Plant Physiology and Ecology, Shanghai Institutes for Biological Sciences, Chinese Academy of Sciences, 200032 Shanghai, China; 2 University of Chinese Academy of Sciences, 100049 Beijing, China; 3 Shanghai Tolo Biotechnology Company Limited, 200233 Shanghai, China; 4 Department of Microbiology and Li KaShing Institute of Health Sciences, The Chinese University of Hong Kong, Prince of Wales Hospital, Shatin, New Territories, Shatin, New Territories China

Correction to: *Cell Research* 10.1038/s41422-018-0022-x, published online 12 March 2018

In the ‘Supplementary information’ file, Figure S1b has been replaced by an updated figure. The labels of crRNA-DNMT-(+4) and crRNA-DNMT-23nt were placed in the wrong order, and the correct order should be “M, control, crRNA-DNMT-(-8), crRNA-DNMT-23nt, crRNA-DNMT-(+4) and crRNA-DNMT-(+8). The order of the crRNA names in the bottom illustrative figure was correct. The authors apologize for this carelessness.

We thank Dr. Panfeng Wu from Hubei University of Science and Technology for pointing out this mistake.

The original article has been corrected.

Original S1b Figure:
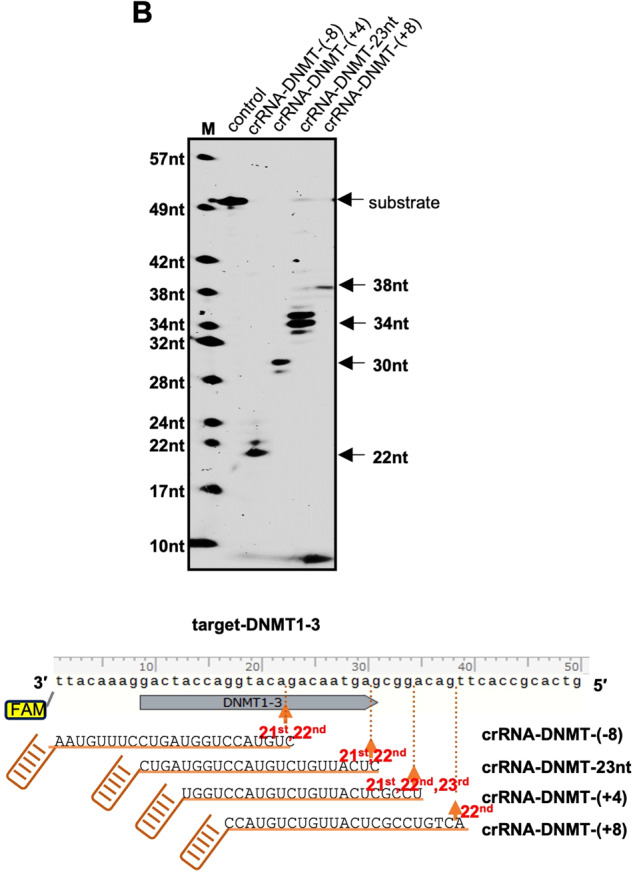


Corrected S1b Figure: