# Supplementary material for: CRISPR-Cas12a has both cis- and trans-cleavage activities on single-stranded DNA
Source: Cell Res. 2018 Mar 12;28(4):491–3. doi: 10.1038/s41422-018-0022-x (PMC5939048; doi:10.1038/s41422-018-0022-x)
Supplement: Supplementary file 1 — Supplementary information [file 41422_2018_22_MOESM1_ESM.pdf]

Supplementary information, Materials and Methods

Generation of dsDNA targets

Short dsDNA targets were prepared through annealing of two complementary oligonucleotides (see Table S2). Paired oligonucleotides (0.8 μM) were annealed in 1×PCR buffer (Transgen Biotech) in a total volume of 50 μL, and then subjected to the annealing program: initial denaturation at 95 °C for 5 min and then cool down from 95 °C to 20 °C with 1 °C decrease per min using a thermocycler.

Transcription of crRNAs

First, the transcription templates were prepared through annealing of the synthesized oligonucleotides with T7-crRNA-F (Table S3), following the same procedures as previously described[1-2]. crRNAs were synthesised using the T7 High Yield Transcription Kit (ThermoFisher Scientific) and the reaction was performed at 37 °C overnight (about 16 h). RNA was purified using the RNA Clean & Concentrator™-5 (Zymo Research) and quantified with NanoDrop 2000C (ThermoFisher Scientific).

Purification of Cas12a proteins

Ten Cas12a coding sequences were codon-optimised and synthesized by Tolo Biotech (Shanghai, China) and then cloned into pET28a (Novagen) with N-terminal 6×His tagging. Oligonucleotides for the construction of site-directed Cas12a mutants are listed in Table S4. For example, to obtain FnCas12a mutants, plasmid pET28a-FnCas12a was amplified by a pair of primers, the products of which were digested with DpnI (1 h, 37 °C) before being transformed into DH10B competent cells. The whole *Cas12a* gene was verified by DNA sequencing before being used for protein expression and purification. Other mutants were obtained following the same procedure.

Cas12a expression plasmids were transformed into *E. coli* BL21 (DE3) (Table S5). For protein expression, a single clone was first cultured overnight in 5-mL liquid LB tubes and then 1% (v/v) inoculated into 3 L of fresh liquid LB. Cells were grown with shaking at 220 rpm and 37 °C until the OD_{600} reached 0.8, and IPTG was then added to a final concentration of 0.2 mM followed by further culture of the cells at 16 °C for about 16 h before the cell harvesting.

Cells were resuspended in 50 mL of lysis buffer [50 mM Tris-HCl (pH 8.0), 1.5 M NaCl, 1 mM DTT and 5% glycerol] with 1 mM PMSF as the protease inhibitor and lysed by high pressure. The obtained lysate was then centrifuged twice at 15,000 rpm for 30 min. After centrifuging, the supernatant was mixed with 5 mL of Ni-NTA beads (GE Healthcare) and softly shaken for 1 h at 4 °C before being loaded onto a 30-mL column. The packing was then washed with wash buffer (lysis buffer supplemented with 30 mM imidazole) and eluted with elution buffer (lysis buffer supplemented with 600 mM imidazole). The elution was dialysed with dialysis buffer 1 [50 mM Tris-HCl (pH 8.0), 200 mM NaCl, 1 mM DTT and 5% glycerol]. Before the protein solution was loaded onto an anion exchange column (HiTrapTM Q HP, GE Healthcare), it was diluted until the final NaCl concentration reached below 80 mM. After that, the column was washed and then eluted with a gradient concentration of NaCl (AKTA Pure, GE Healthcare). Fractions containing Cas12a proteins were verified by SDS-PAGE and then pooled for dialysis with dialysis buffer 2 [20 mM Tris-HCl (pH 8.0), 600 mM NaCl, 2 mM DTT, 0.2 mM EDTA] overnight. Finally, the protein was collected and diluted to a final concentration of 6 μM and mixed with an equal volume of 100% cold glycerol prior to being frozen at −80 °C.

***In vitro* cleavage assay**

Unless mentioned otherwise, FnCas12a was used for the cleavage assays in this study. For the cleavage of target plasmid, the *in vitro* cleavage system contained 500 ng of target DNA, 250 nM Cas12a and 500 nM synthesised crRNA in a 20-μL reaction system. The reaction was performed at 37 °C in NEB buffer 3 for 1 h. For cleavage of M13mp18 by Cas12a, the reaction was performed at 37 °C in NEB buffer 3 for 30 min, employing 100 nM Cas12a, 250 nM synthesised crRNA, 1 μg M13mp18 DNA , 40 nM *trans* ssDNA and 10 U RNase inhibitor (Takara) in a 20-μL volume. For cleavage of M13mp18 by exonuclease T (NEB), the reaction was incubated at 25 °C for 30 min in NEB buffer 4.

Cleavage of FAM-labelled single-stranded oligonucleotides or double-stranded oligonucleotides was performed at 37 °C in NEB buffer 3 for 1 h, employing 250 nM Cas12a, 500 nM synthesised crRNA, 40 nM target DNA or/and 40 nM *trans* ssDNA and 10 U RNase inhibitor (Takara) in a 20-μL volume. Reactions were stopped by heating at 98 °C for 10 min, followed by immediately chilling on ice before further analysis through urea PAGE. For the time-course experiments in Supplementary information Figure S2a, the final

concentration of crRNA and Cas12a was reduced to 100 nM.

For LC-MS detection, cleavage of samples was performed in NEB buffer 3 at 37 °C for 2 h, containing 1 μM FnCas12a, 1 μM synthesised crRNA-T1, 1 μM target DNA (target-T1-18), 25 μM *trans* ssDNA (10T-FAM-5′ or 10T-FAM-3′) and 10 U RNase inhibitor.

Analysis of Cas12a-digested products by denaturing urea PAGE or LC-MS

To analyze the products by urea PAGE, FAM-labelled DNA was first digested by Cas12a and then heated at 98 °C for 10 min after the addition of loading buffer to stop the reactions. Heated samples were immediately chilled on ice, followed by being loaded on 10%–15% denaturing polyacrylamide gels containing 7 M urea. Electrophoresis was performed by running at 1800 V (about 40 V/cm) for about 70–90 min (using the Sequi-Gen GT Sequencing Cell system, Bio-Rad) or at 200 V (about 40 V/cm) for about 25 min (using the Mini-PROTEAN Tetra Cell system, Bio-Rad) in 1× TBE buffer. Gels were scanned using a FLA-9000 phosphoimager (FujiFilm Corporation, Japan).

For LC-MS analysis, FnCas12a-digested samples were first de-salted using a Novatia Oligo HTCS trap column (C18 300A, 1×10 mm), and were then analyzed with a Thermo LCQ ion trap with an ESI source (negative ion mode), where the electrospray gas was heated to about 300 °C. The liquid phase conditions are HFIPA-based aqueous reagents, and the system was operated with a flow of 40% ACN mixed with 40% MeOH and 20% water.

Supplementary information, Table S1 Sequences of Cas12a proteins used in this study

Name	GI number	Species
FnCas12a	489130501	*Francisella tularensis*
AsCas12a	545612232	*Acidaminococcus sp.* BV3L6
LbCas12a	917059416	*Lachnospiraceae bacterium* ND2006
Lb5Cas12a	652820612	*Lachnospiraceae bacterium* NC2008
HkCas12a	491540987	*Helcococcus kunzii* ATCC 51366
OsCas12a	909652572	*Oribacterium sp.* NK2B42
TsCas12a	972924080	*Thiomicrospira sp.* XS5
BbCas12a	987324269	*Bacteroidales bacterium* KA00251
BoCas12a	496509559	*Bacteroidetes oral taxon* 274 str. F0058
Lb4Cas12a	769130406	*Lachnospiraceae bacterium* MC2017

Supplementary information, Table S2 Oligonucleotides used for preparation of cleavage templates in this study

Oligo names	Sequences (5′-3′)
target-DNMT1-3-F	aatgtttcctgatggtccatgtctgttactcgcctgtcaagtggcgtgac
target-DNMT1-3-R	gtcacgccacttgacaggcgagtaacagacatggaccatcaggaaacatt
target-DNMT1-3-R-FAM-3′	gtcacgccacttgacaggcgagtaacagacatggaccatcaggaaacatt-FAM
target-DNMT1-3-R-FAM-5′	FAM-gtcacgccacttgacaggcgagtaacagacatggaccatcaggaaacatt
target-DNMT1-3-F-FAM-3′	aatgtttcctgatggtccatgtctgttactcgcctgtcaagtggcgtgac-FAM
target-DNMT1-3-F-FAM-5′	FAM-aatgtttcctgatggtccatgtctgttactcgcctgtcaagtggcgtgac
target-T1-F	tttctgtttgttatcgcaactttctactgaattcaagctttactctagaaagaggagaaaggatcc
target-T1-R	ggatcctttctcctctttctagagtaaagcttgaattcagtagaaagttgcgataacaaacagaaa
target-T1-F-FAM	FAM-tttctgtttgttatcgcaactttctactgaattcaagctttactctagaaagaggagaaaggatcc
target-T1-R-FAM	ggatcctttctcctctttctagagtaaagcttgaattcagtagaaagttgcgataacaaacagaaa-FAM
target-T1-FAM-3′-F	tttctgtttgttatcgcaactttctactgaattcaagctttactctagaaagaggagaaaggatcc-FAM
target-T1-FAM-5′-R	FAM-ggatcctttctcctctttctagagtaaagcttgaattcagtagaaagttgcgataacaaacagaaa
target-DNMT1-3-R-TTT-FAM-3′	gtcacgccacttgacaggcgagtaacagacatggaccatcaggTTTcatt-FAM
target-DNMT1-3-R-CCC-FAM-3′	gtcacgccacttgacaggcgagtaacagacatggaccatcaggCCCcatt-FAM
target-DNMT1-3-F-AAA	aatgAAAcctgatggtccatgtctgttactcgcctgtcaagtggcgtgac
target-DNMT1-3-F-GGG	aatgGGGcctgatggtccatgtctgttactcgcctgtcaagtggcgtgac
target-T1-1-R	acaaacagaaa
target-T1-6-R	cgataacaaacagaaa
target-T1-12-R	aagttgcgataacaaacagaaa
target-T1-18-R	agtagaaagttgcgataacaaacagaaa
target-T1-24-R	gaattcagtagaaagttgcgataacaaacagaaa
target-T1-24-only-R	gaattcagtagaaagttgcgataa
target-T1-18-only-R	agtagaaagttgcgataa
target-T1-12-only-R	aagttgcgataa
target-T1-6-only-R	cgataa
N25-5′FAM	FAM-NNNNNNNNNNNNNNNNNNNNNNNNN
N25-3′FAM	NNNNNNNNNNNNNNNNNNNNNNNNN-FAM
Target-T2	ctagagtaaagcttgaattcagtagaaa
Target-T3	ggatccactagtctctagctcgagaaa
Target-T4	ttcaaggagaaactgcagctagcttaaa
target-DNMT1-3-R-FAM-5′-JOE-3′	FAM-gtcacgccacttgacaggcgagtaacagacatggaccatcaggaaacatt-JOE
10T-FAM-5′	FAM-TTTTTTTTTT
10T-FAM-3′	TTTTTTTTTT-FAM

Supplementary information, Table S3 Oligonucleotides used for preparation of transcription templates in this study

Oligo names	Sequences (5′-3′)
T7-crRNA-F	GAAATTAATACGACTCACTATAGGG
T7-T1-24-R	gaattcagtagaaagttgcgataaATCTACAACAGTAGAAATTCCCTATAGTGAGTCGTATTAATTTC
T7-T1-8-R	tgcgataaATCTACAACAGTAGAAATTCCCTATAGTGAGTCGTATTAATTTC
T7-T1-10-R	gttgcgataaATCTACAACAGTAGAAATTCCCTATAGTGAGTCGTATTAATTTC
T7-T1-12-R	aagttgcgataaATCTACAACAGTAGAAATTCCCTATAGTGAGTCGTATTAATTTC
T7-T1-14-R	gaaagttgcgataaATCTACAACAGTAGAAATTCCCTATAGTGAGTCGTATTAATTTC
T7-T1-15-R	agaaagttgcgataaATCTACAACAGTAGAAATTCCCTATAGTGAGTCGTATTAATTTC
T7-T1-16-R	tagaaagttgcgataaATCTACAACAGTAGAAATTCCCTATAGTGAGTCGTATTAATTTC
T7-T1-17-R	gtagaaagttgcgataaATCTACAACAGTAGAAATTCCCTATAGTGAGTCGTATTAATTTC
T7-T1-18-R	agtagaaagttgcgataaATCTACAACAGTAGAAATTCCCTATAGTGAGTCGTATTAATTTC
T7-T1-20-R	tcagtagaaagttgcgataaATCTACAACAGTAGAAATTCCCTATAGTGAGTCGTATTAATTTC
T7-T1-22-R	attcagtagaaagttgcgataaATCTACAACAGTAGAAATTCCCTATAGTGAGTCGTATTAATTTC
T7-T1-30-R	aagcttgaattcagtagaaagttgcgataaATCTACAACAGTAGAAATTCCCTATAGTGAGTCGTATTAATTTC
T7-crRNA-DNMT-23nt-R	GAGTAACAGACATGGACCATCAGATCTACAACAGTAGAAATTCCCTATAGTGAGTCGTATTAATTTC
T7-crRNA-DNMT-(-8)-R	gacatggaccatcaggaaacattATCTACAACAGTAGAAATTCCCTATAGTGAGTCGTATTAATTTC
T7-crRNA-DNMT-(+4)-R	aggcgagtaacagacatggaccaATCTACAACAGTAGAAATTCCCTATAGTGAGTCGTATTAATTTC
T7-crRNA-DNMT-(+8)-R	tgacaggcgagtaacagacatggATCTACAACAGTAGAAATTCCCTATAGTGAGTCGTATTAATTTC
T7-crRNA-DNMT-16nt-R	agacatggaccatcagATCTACAACAGTAGAAATTCCCTATAGTGAGTCGTATTAATTTC
T7-crRNA-DNMT-18nt-R	acagacatggaccatcagATCTACAACAGTAGAAATTCCCTATAGTGAGTCGTATTAATTTC
T7-crRNA-DNMT-20nt-R	taacagacatggaccatcagATCTACAACAGTAGAAATTCCCTATAGTGAGTCGTATTAATTTC
T7-T2-R	ctagagtaaagcttgaattcagtaATCTACAACAGTAGAAATTCCCTATAGTGAGTCGTATTAATTTC
T7-T3-R	ggatccactagtctctagctcgaATCTACAACAGTAGAAATTCCCTATAGTGAGTCGTATTAATTTC
T7-T4-R	ttcaaggagaaactgcagctagctATCTACAACAGTAGAAATTCCCTATAGTGAGTCGTATTAATTTC

Supplementary information, Table S4 Oligonucleotides used for plasmids construction in this study

Oligo names	Sequences (5′-3′)
FnCas12a-K869A-F	gcttgtctgcgatcagatcgtactcgaacacg
FnCas12a-K869A-R	atctgatcgcagacaagcggttcaccgaggataag
FnCas12a-K852A-F	tgtccttatttgcgttggcgatggcctccttggc
FnCas12a-K852A-R	atcgccaacgcaaataaggacaatcctaagaagg
FnCas12a-H843A-F	ttggctggtgctgtgatcttcttagggatggac
FnCas12a-H843A-R	agaagatcacagcaccagccaaggaggccatcg

FnCas12a-R1218A-F	ggtcttgctgttggccatctgcaggattgtattc
FnCas12a-R1218A-R	tcctgcagatggccaacagcaagaccggcacagagc
FnCas12a-E1006A-F	agttcagatctgcgaacaccacgatggcattg
FnCas12a-E1006A-R	tcgtggtgttcgcagatctgaacttcggctttaagag
FnCas12a-D917A-F	tctcgccccgtgcgatgctcaggatgtgcacatcg
FnCas12a-D917A-R	tcctgagcatcgcacggggcgagagacacctgg
FnCas12a-D1255A-F	gcgccgttggctgcggcatcctgaggcatattcttgg
FnCas12a-D1255A-R	ctcaggatgccgcagccaacggcgcctatcacatcggcctg

Supplementary information, Table S5 Plasmids and strains used in this study

Plasmids or Strains	Relevant properties or genotypes	Sources
**Plasmids**		
pET28a-TEV	pET28a with the thrombin cleavage site changed to the TEV protease cleavage site	[3]
pET28a-TEV-FnCas12a	pET28a-TEV carrying FnCas12a	[1]
pET28a-TEV-AsCas12a	pET28a-TEV carrying AsCas12a	[2]
pET28a-TEV-LbCas12a	pET28a-TEV carrying LbCas12a	[2]
pET28a-TEV-Lb5Cas12a	pET28a-TEV carrying Lb5Cas12a	This study
pET28a-TEV-HkCas12a	pET28a-TEV carrying HkCas12a	This study
pET28a-TEV-OsCas12a	pET28a-TEV carrying OsCas12a	This study
pET28a-TEV-TsCas12a	pET28a-TEV carrying TsCas12a	This study
pET28a-TEV-BbCas12a	pET28a-TEV carrying BbCas12a	This study
pET28a-TEV-BoCas12a	pET28a-TEV carrying BoCas12a	This study
pET28a-TEV-Lb4Cas12a	pET28a-TEV carrying Lb4Cas12a	This study
pET28a-TEV-FnCas12a-K869A	pET28a-TEV carrying FnCas12a-K869A	This study
pET28a-TEV-FnCas12a-K852A	pET28a-TEV carrying FnCas12a-K852A	This study
pET28a-TEV-FnCas12a-H843A	pET28a-TEV carrying FnCas12a-H843A	This study
pET28a-TEV-FnCas12a-R1218A	pET28a-TEV carrying FnCas12a-R1218A	This study
pET28a-TEV-FnCas12a-E1006A	pET28a-TEV carrying FnCas12a-E1006A	This study
pET28a-TEV-FnCas12a-D917A	pET28a-TEV carrying FnCas12a-D917A	This study
pET28a-TEV-FnCas12a-D1255A	pET28a-TEV carrying FnCas12a-D1255A	This study
M13mp18 single-stranded DNA		NEB
pCB1A2_2		[2]
**Strains**		
E. coli DH10B	F^- *endA1* $deoR^+$ *recA1 galE15 galK16 nup G rpsL* Δ*(lac)X74* φ80*lacZΔM15 araD139* Δ*(ara,leu)7697 mcrA* Δ*(mrr-hsdRMS-mcrBC)* Str^R λ^-	Invitrogen
E. coli BL21(DE3)	F– *ompT gal dcm lon hsdSB(rB- mB-) λ(DE3* [*lacI lacUV5-T7 gene 1 ind1 sam7 nin5*])	Invitrogen

Supplementary information, nucleotide sequences used in this study

>FnCas12a (codon-optimised DNA sequence)

atgagcatctatcaggagttcgtgaataagtacagcctgtccaagaccctgcggtttgagctgatcccccagggcaagacactggagaacatcaaggccagggg
cctgatcctggacgatgagaagcgcgccaaggactataagaaggccaagcagatcatcgataagtaccaccagttctttatcgaggagatcctgagcagcgtgtg
catctctgaggatctgctgcagaattacagcgacgtgtatttcaagctgaagaagtctgacgatgacaacctgcagaaggacttcaagagcgccaaggacaccatc
aagaagcagatcagcgagtatatcaaggactccgagaagtttaagaatctgttcaaccagaatctgatcgatgccaagaagggccaggagtccgacctgatcctgt
ggctgaagcagtctaaggacaatggcatcgagctgttcaaggccaactctgatatcaccgatatcgacgaggccctggagatcatcaagagctttaagggctgga
ccacatactttaagggcttccacgagaacaggaagaacgtgtacagcagcaacgacatccctacaagcatcatctaccgcatcgtggatgacaatctgccaaagtt
cctggagaacaaggccaagtatgagtccctgaaggacaaggcccccgaggccatcaattacgagcagatcaagaaggatctggccgaggagctgaccttcgat
atcgactataagacatccgaggtgaaccagcgggtgttttctctggacgaggtgtttgagatcgccaatttcaacaattacctgaaccagtccggcatcaccaagttc
aatacaatcatcggcggcaagtttgtgaacggcgagaataccaagagaaagggcatcaacgagtacatcaatctgtatagccagcagatcaacgacaagaccct
gaagaagtacaagatgagcgtgctgttcaagcagatcctgtccgatacagagtctaagagctttgtgatcgataagctggaggatgactctgacgtggtgaccaca
atgcagagcttttatgagcagatcgccgccttcaagaccgtggaggagaagtctatcaaggagacactgagcctgctgttcgatgacctgaaggcccagaagctg
gacctgtctaagatctacttcaagaacgataagtccctgaccgacctgtctcagcaggtgtttgatgactatagcgtgatcggcaccgccgtgctggagtacatcaca
cagcagatcgccccaaagaacctggataatccctctaagaaggagcaggagctgatcgccaagaagaccgagaaggccaagtatctgagcctggagacaatca
agctggccctggaggagttcaataagcaccgggatatcgacaagcagtgcagatttgaggagatcctggccaacttcgccgccatccccatgatctttgatgagat
cgcccagaacaaggacaatctggcccagatctccatcaagtaccagaaccagggcaagaaggacctgctgcaggcctctgccgaggatgacgtgaaggccat
caaggatctgctggaccagaccaacaatctgctgcacaagctgaagatcttccacatctcccagtctgaggataaggccaatatcctggataaggacgagcactttt
atctggtgttcgaggagtgttacttcgagctggccaacatcgtgcccctgtacaacaagatcagaaattatatcacacagaagccttactccgacgagaagtttaagc
tgaacttcgagaacagcaccctggccaacggctgggataagaataaggagcctgacaacacagccatcctgttcatcaaggatgacaagtactatctgggcgtga
tgaataagaagaacaataagatcttcgatgacaaggccatcaaggagaacaagggcgagggctacaagaagatcgtgtataagctgctgcccggcgccaataa
gatgctgcctaaggtgttcttttccgccaagtctatcaagttctacaacccatccgaggacatcctgcggatcagaaatcactccacccacacaaagaacggctctcc
ccagaagggctatgagaagtttgagttcaatatcgaggattgccggaagtttatcgacttctacaagcagagcatctccaagcaccctgagtggaaggattttggctt
caggtttagcgacacccagcggtacaactccatcgacgagttctacagagaggtggagaatcagggctataagctgacatttgagaacatctctgagagctacatc
gacagcgtggtgaatcagggcaagctgtacctgttccagatctataacaaggacttcagcgcctattccaagggccggccaaacctgcacaccctgtactggaag
gccctgttcgatgagagaaatctgcaggacgtggtgtataagctgaacggcgaggccgagctgttttacaggaagcagtccatccctaagaagatcacacaccca
gccaaggaggccatcgccaacaagaataaggacaatcctaagaaggagagcgtgttcgagtacgatctgatcaaggacaagcggttcaccgaggataagttcttt
ttccactgtccaatcacaatcaacttcaagtcctctggcgccaacaagtttaatgacgagatcaatctgctgctgaaggagaaggccaacgatgtgcacatcctgag
catcgaccggggcgagagacacctggcctactataccctggtggatggcaagggcaatatcatcaagcaggataccttcaacatcatcggcaatgacaggatga
agacaaactaccacgataagctggccgccatcgagaaggatagggactccgcccgcaaggactggaagaagatcaacaatatcaaggagatgaaggagggct
atctgtctcaggtggtgcacgagatcgccaagctggtcatcgagtacaatgccatcgtggtgttcgaggatctgaacttcggctttaagaggggccgctttaaggtg
gagaagcaggtgtatcagaagctggagaagatgctgatcgagaagctgaattacctggtgtttaaggataacgagttcgacaagaccggaggcgtgctgagggc
ataccagctgaccgcccccttttgagacattcaagaagatgggcaagcagacaggcatcatctactatgtgccagccggcttcacctccaagatctgccccgtgaca
ggctttgtgaaccagctgtaccctaagtatgagtccgtgtctaagagccaggagtttttcagcaagttcgataagatctgttataatctggacaagggctacttcgagtt
ttccttcgattataagaactttggcgacaaggccgccaagggcaagtggaccatcgcctctttcggcagccggctgatcaactttagaaattccgataagaaccaca
attgggacacccgggaggtgtacccaacaaaggagctggagaagctgctgaaggactacagcatcgagtatggccacggcgagtgcatcaaggccgccatct
gtggcgagagcgataagaagtttttcgccaagctgacctccgtgctgaatacaatcctgcagatgcggaacagcaagaccggcacagagctggactacctgatct
cccccgtggccgatgtgaacggcaacttcttcgacagcagacaggcccccaagaatatgcctcaggatgccgacgccaacggcgcctatcacatcggcctgaa
gggcctgatgctgctgggcaggatcaagaacaatcaggagggcaagaagctgaacctggtcatcaagaacgaggagtactttgagttcgtgcagaaccgcaac
aattga

>AsCas12a (codon-optimised DNA sequence)

atgacacagttcgagggctttaccaacctgtatcaggtgagcaagacactgaggtttgagctgatcccacagggcaagaccctgaagcacatccaggagcaggg
cttcatcgaggaggacaaggccagaaatgatcactacaaggagctgaagcccatcatcgataggatctacaagacctatgccgaccagtgcctgcagctggtgc
agctggattgggagaacctgagcgccgccatcgactcctatcgcaaggagaagacagaggagacccggaacgccctgatcgaggagcaggccacatatagaa
atgccatccacgactacttcatcggcaggacagacaacctgaccgatgccatcaataagcgccacgccgagatctacaagggcctgttcaaggccgagctgttta
atggcaaggtgctgaagcagctgggcaccgtgaccacaaccgagcacgagaacgccctgctgcgcagcttcgataagtttacaacctacttctccggcttttatga
gaaccggaagaacgtgttcagcgccgaggatatcagcacagccatcccacacagaatcgtgcaggacaacttccccaagtttaaggagaattgtcacatcttcac
aagactgatcaccgccgtgcccagcctgagggagcactttgagaacgtgaagaaggccatcggcatcttcgtgagcacctccatcgaggaggtgttttccttccct
ttttataaccagctgctgacacagacccagatcgacctgtacaatcagctgctgggaggaatctctagggaggcaggaaccgagaagatcaagggcctgaacga
ggtgctgaatctggccatccagaagaatgatgagacagcccacatcatcgcctccctgccacaccgcttcatcccccctgtttaagcagatcctgtccgatcggaaca
ccctgtctttcatcctggaggagtttaagagcgacgaggaagtgatccagtccttctgcaagtacaagacactgctgcgcaacgagaatgtgctggagaccgccga
ggccctgtttaacgagctgaacagcatcgacctgacacacatcttcatcagccacaagaagctggagaccatcagcagcgccctgtgcgaccactgggatacact
gcggaatgccctgtacgagcggagaatctccgagctgacaggcaagatcaccaagtctgccaaggagaaggtgcagagaagcctgaagcacgaggatatcaa
cctgcaggagatcatctctgccgcaggcaaggagctgagcgaggccttcaagcagaagaccagcgagatcctgtcccacgcacacgccgccctggatcagcc
actgcctacaaccctgaagaagcaggaggagaaggagatcctgaagtctcagctggacagcctgctgggcctgtaccacctgctggactggtttgccgtggatg
agtccaacgaggtggaccccgagttctctgccaggctgaccggcatcaagctggagatggagccttctctgagcttctacaacaaggcccgcaattatgccacca
agaagccctactccgtggagaagttcaagctgaactttcagatgcctacactggcctctggctgggacgtgaataaggagaagaacaatggcgccatcctgtttgt
gaagaacggcctgtactatctgggcatcatgccaaagcagaagggcaggtataaggccctgagcttcgagcccacagagaagacctccgagggctttgataaga
tgtactatgactacttccctgatgccgccaagatgatcccaaagtgcagcacccagctgaaggccgtgacagcccactttcagacccacacaacccccatcctgct
gtccaacaatttcatcgagcctctggagatcacaaaggagatctacgacctgaacaatcctgagaaggagccaaagaagtttcagacagcctatgccaagaagac
cggcgatcagaagggatacagggaggccctgtgcaagtggatcgacttcacacgggattttctgtccaagtataccaagacaacctctatcgatctgtctagcctga
ggccatcctctcagtataaggacctgggcgagtactatgccgagctgaatcccctgctgtaccacatcagcttccagcgcatcgccgagaaggagatcatggatg
ccgtggagaccggcaagctgtacctgttccagatctataacaaggactttgccaagggccaccacggcaagcctaatctgcacacactgtattggaccggcctgtt
ttctccagagaacctggccaagacaagcatcaagctgaatggccaggccgagctgttctacagacctaagtccaggatgaagcgcatggcccacaggctgggc
gagaagatgctgaacaagaagctgaaggatcagaagacaccaatccccgacaccctgtaccaggagctgtacgactatgtgaatcaccgcctgtcccacgacct
gtctgatgaggcccgggccctgctgccaaacgtgatcaccaaggaggtgtctcacgagatcatcaaggataggcgctttaccagcgacaagttctttttccacgtg
cctatcacactgaactatcaggccgccaattccccatctaagttcaaccagcgggtgaatgcctacctgaaggagcaccccgagacccctatcatcggcatcgatc
ggggcgagagaaacctgatctatatcacagtgatcgactccaccggcaagatcctggagcagagatctctgaataccatccagcagtttgattaccagaagaagct
ggacaaccgggagaaggagagagtggcagcaaggcaggcctggtctgtggtgggcacaatcaaggatctgaagcagggctatctgagccaggtcatccacga
gatcgtggacctgatgatccactaccaggccgtggtggtgctggagaacctgaatttcggctttaagagcaagaggaccggaatcgcagagaaggccgtgtacc
agcagttcgagaagatgctgatcgataagctgaattgcctggtgctgaaggactatccagcagagaaagtgggaggcgtgctgaacccataccagctgacagac
cagttcacctcctttgccaagatgggcacccagtctggcttcctgttttacgtgcctgccccatatacatctaagatcgatcccctgaccggcttcgtggaccccttcgt
gtggaagaccatcaagaatcacgagagcagaaagcacttcctggagggcttcgactttctgcactacgatgtgaagacaggcgacttcatcctgcactttaagatg
aaccgcaatctgtccttccagaggggactgccaggctttatgcctgcatgggatatcgtgttcgagaagaacgagacacagtttgacgccaagggcacccctttcat
cgccggcaagcgcatcgtgccagtgatcgagaatcaccggtttaccggccggtacagagacctgtatcctgccaacgagctgatcgccctgctggaggagaag
ggcatcgtgttcagagatggctccaacatcctgccaaagctgctggagaatgacgattctcacgccatcgacacaatggtggccctgatcagaagcgtgctgcag
atgaggaactccaatgccgccacaggcgaggactatatcaacagccccgtgagagatctgaatggcgtgtgcttcgactccaggtttcagaacccagagtggcct
atggacgccgatgccaatggcgcctaccacatcgccctgaagggccagctgctgctgaatcacctgaaggagagcaaggatctgaagctgcagaacggcatct
ccaatcaggactggctggcctacatccaggagctgaggaactag

>LbCas12a (codon-optimised DNA sequence)

atgctgaagaacgtgggcatcgacagactggatgtggagaagggcagaaagaacatgagcaagctggagaagttcaccaactgctacagcctgagcaagacc
ctgagattcaaggccatccccgtgggaaaaacccaggagaacatcgacaacaagagactgctggtggaggacgaaaagagagccgaggactacaagggcgt

gaagaagctgctggacagatactacctgagcttcatcaacgacgtgctgcacagcatcaagctgaagaacctgaacaactacatcagcctgttcagaaagaagac
cagaaccgagaaggagaacaaggagctggagaacctggagatcaacctgagaaaggagatcgccaaggccttcaagggaaacgagggctacaagagcctgt
tcaagaaggacatcatcgagaccatcctgcccgagttcctggatgacaaggacgagatcgccctggtgaacagcttcaacggcttcaccaccgctttcaccggctt
cttcgacaacagagagaacatgttcagcgaggaggccaagtctacaagcatcgccttcagatgcatcaacgagaacctgaccagatacatcagcaacatggacat
cttcgagaaggtggacgccatcttcgacaagcacgaggtgcaggagatcaaggagaagatcctgaacagcgactacgacgtggaggacttcttcgagggcgag
ttcttcaacttcgtgctgacccaggaaggcatcgacgtgtacaacgccatcatcggcggatttgtgacagagagcggcgagaaaatcaagggcctgaacgagtac
atcaacctgtacaaccagaagaccaagcagaagctgcccaagttcaagcccctgtacaagcaggtgctgagcgacagagagagcctgagcttctatggcgagg
gctacaccagcgatgaagaggtgctggaggtgttcagaaacaccctgaacaagaacagcgagatcttcagcagcatcaagaagctggagaagctgttcaagaa
cttcgacgagtacagcagcgccggcatctttgtgaaaaacggccccgctatcagcacaatcagcaaggacatcttcggcgagtggaacgtgatcagagacaagt
ggaacgccgagtacgacgacatccacctgaagaagaaggccgtggtgaccgagaaatacgaggacgacagaagaaagagcttcaagaagatcggcagcttc
agcctggaacagctgcaagagtacgctgacgctgacctgagcgttgtggagaagctgaaggagatcatcatccagaaggtggacgagatctacaaggtgtacg
gcagcagcgagaaacttttcgacgccgacttcgtgcttgagaagagcctgaagaagaacgatgccgtggtggccatcatgaaggacctgctggacagcgtgaag
agcttcgagaactacatcaaggccttcttcggcgaaggcaaggagaccaacagagacgagagcttctacggcgacttcgtgctggcttacgacatcctgctgaag
gtggaccacatctacgacgccatcagaaactacgtgacccagaagccctacagcaaggacaagttcaagctgtacttccagaacccccagtttatgggcggatgg
gacaaggataaggagaccgactacagagccaccatcctgagatacggcagcaagtactacctggccatcatggacaagaagtacgccaagtgcctgcagaaga
tcgacaaggacgacgtgaacggcaactacgagaagatcaactacaagctgctgcccggccctaataaaatgctgcccaaggtgttcttcagcaagaagtggatg
gcctactacaaccccagcgaggacatccagaagatctacaagaacggcaccttcaagaagggcgacatgttcaacctgaacgactgccacaagctgatcgactt
cttcaaggacagcatcagcagataccccaagtggagcaacgcctacgacttcaacttcagcgagaccgagaagtacaaggacatcgccggcttctacagagaag
tggaggagcagggatacaaggtgagcttcgagagcgccagcaagaaggaggtggacaagctggtggaagagggcaagctgtacatgttccagatctacaaca
aggacttcagcgacaagtctcacggaacccccaatctgcacaccatgtacttcaagctgctgttcgacgagaacaaccacggccagatcagactttctggaggcg
ctgaactgttcatgagaagagccagcctgaagaaggaagagctggtggtgcatcctgccaatagccccatcgctaacaagaaccccgacaaccccaagaaaac
caccaccctgagctacgacgtgtacaaggacaagagattcagcgaggaccagtacgagctgcatatccccatcgccatcaacaagtgccccaagaacatcttca
agatcaacaccgaggtgagagtgctgctgaagcacgacgacaacccctacgtgatcggcattgacagaggcgagagaaacctgctgtacatcgtggtggtgga
cggcaagggaaacatcgtggagcagtacagcctgaacgagatcatcaacaacttcaacggcatcagaatcaagaccgactaccacagcctgctggacaagaag
gagaaggagagattcgaggccagacagaactggaccagcatcgagaacatcaaggagctgaaggccggctacattagccaggtggtgcacaagatctgcgag
ctggtggagaagtacgatgccgtgatcgctctggaggatctgaacagcggcttcaagaacagcagagtgaaggtggagaagcaggtgtaccagaagttcgaga
agatgctgatcgacaagctgaactacatggtggacaagaagagcaacccctgtgctacaggcggagctctgaagggataccagatcaccaacaagttcgagag
cttcaagagcatgagcacccagaacggcttcatcttctacatccccgcctggctgacatctaagatcgaccctagcaccggctttgtgaacctgctgaagaccaagt
acaccagcatcgccgacagcaagaagttcatcagcagcttcgacagaatcatgtacgtgcccgaggaggacctgtttgaatttgccctggactacaagaacttcag
cagaaccgacgccgactacatcaagaagtggaagctgtacagctacggcaacagaatcagaatcttcagaaaccccaagaagaacaacgtgttcgactgggag
gaggtgtgtctgacaagcgcctacaaggagctgttcaacaagtacggcatcaactaccagcagggcgacattagagccctgctgtgcgaacagagcgacaagg
ccttctacagcagcttcatggccctgatgagcctgatgctgcagatgagaaacagcatcaccggcagaaccgacgtggacttccttatcagccccgtgaaaaaca
gcgacggcatcttctacgacagcagaaactacgaggcccaggagaatgctatcctgcccaagaatgccgatgctaacggcgcttacaacatcgccagaaaggtg
ctttgggccatcggccagtttaagaaggccgaggacgagaagctggacaaggtgaagatcgccatcagcaacaaggagtggctggagtatgctcagaccagcg
tgaaacactga

>Lb5Cas12a (codon-optimised DNA sequence)

atggagaactactacgacagcctgaccagacaataccccgtgaccaagaccatcagacaggagctgaagcccgtgggaaaaaccctggagaacatcaagaac
gccgagatcatcgaggccgacaagcagaagaaggaggcctacgtgaaggtgaaggagctgatggacgagttccacaagagcatcatcgagaagagcctggt
gggcattaaactggacggcctgagcgaatttgagaagctgtacaagatcaagaccaagaccgacgaggacaagaacagaatcagcgagctgttctactacatga
gaaagcagatcgccgacgccctgaagaacagcagagactacggctacgtggacaacaaggacctgatcgagaagatcctgcccgagagagtgaaggacgag
aacagcctgaacgccctgagctgcttcaaaggcttcaccacctacttcaccgactactacaagaacaggaagaacatctacagcgacgaggagaagcatagcac

cgtgggctacagatgcatcaacgagaacctgctgatcttcatgagcaacatcgaggtgtaccagatctacaagaaggccaacatcaagaacgacaactacgacg
aggagaccctggacaagaccttcatgatcgagagcttcaacgagtgcctgacccaaagcggcgtggaggcttacaatagcgtggtggccagcattaagacagcc
accaacctgtacatccagaagaacaacaaggaggagaacttcgtgagagtgcccaagatgaaggtgctgttcaagcagatcctgagcgacagaaccagccttttc
gacggcctgatcatcgaaagcgacgacgagctgctggataagctgtgcagcttcagcgctgaggtggacaagttcctgcccatcaacatcgacagatacatcaag
accctgatggacagcaataacggcaccggcatctacgtgaagaacgacagcagcctgaccaccctgagcaactacctgaccgacagctggagcagcatcaga
aacgccttcaacgagaactacgacgccaagtacaccggcaaggtgaacgacaagtacgaggagaagagagagaaggcctacaagagcaacgacagcttcga
gctgaactacatccagaacctgctgggcatcaacgtgatcgacaagtacatcgagagaatcaacttcgacatcaaggagatctgcgaggcctacaaggagatgac
caagaactgcttcgaggaccacgacaagaccaagaagctgcagaagaacatcaaggccgtggccagcatcaagagctacctggacagcctgaagaacatcga
gagagacatcaagctgctgaacggcacaggactggagagcagaaacgagttcttctacggcgagcagagcacagttctggaggagatcaccaaggtggacga
gctgtacaacatcaccagaaactacctgaccaagaagcccttcagcaccgagaagatgaagctgaacttcaacaacccccagctgctgggaggatgggacgtga
acaaggagagagactgctacggcgtgatcctgatcaaggacaacaactactacctgggcatcatggacaagagcgccaacaagagcttcctgaacatcaagga
gagcaagaacgagaacgcctacaagaaggtgaactgcaagctgctgcccggacccaataagatgttccccaaggtgttcttcgccaagagcaacatcgactact
acgaccccacccacgagatcaagaagctgtacgacaagggcaccttcaagaagggcaacagcttcaacctggaggactgccacaagctgatcgacttctacaa
ggagagcatcaagaagaacgacgactggaagaacttcaacttcaacttcagcgacaccaaggactacgaggacatcagcggcttcttcagagaagtggaggcc
cagaactacaagatcacctacaccaacgtgagctgcgacttcatcgagagcctggtggatgagggcaagctgtacctgttccagatctacaacaaggacttcagc
gagtacgctaccggcaacctgaatctgcacaccctgtacctgaagatgctgttcgacgagagaaacctgaaggacctgtgcatcaagatgaacggcgaggccga
ggtgttttatagacccgccagcattctggatgaggacaaggtggtgcacaaggccaaccagaagatcaccaacaagaacaccaacagcaagaagaaggagag
catcttcagctacgacatcgtgaaggacaagagatacaccgtggacaagttcttcatccacctgcccatcaccctgaactacaaggagcagaacgtgagcagattc
aacgactacatcagagagatcctgaagaagagcaagaacatcagagtgatcggcatcgacagaggcgagagaaacctgctgtacgtggtggtgtgcgatagcg
atggcagcatcctgtaccagagaagcatcaacgagatcgtgagcggcagccacaaaaccgactaccacaagctgctggacaacaaggagaaggagagactga
gcagcagaagagactggaagaccatcgagaacatcaaggacctgaaggccggctacatgagccaagtggtgaacgagatctacaacctgatcctgaagtacaa
cgccatcgtggtgctggaggacctgaacatcggcttcaagaacggcagaaagaaggtggagaagcaggtgtaccagaacttcgagaaggccctgatcgacaa
gctgaactacctgtgcatcgacaagaccagagagcagctgtctccttctagccctggaggagtgctgaacgcctatcagctgaccgctaagttcgagagcttcgag
aagatcggcaagcagaccggctgcatcttctatgtgcccgcctacctgacatctcagattgaccccacaaccggcttcgtgaacctgttctaccagaaggacacca
gcaaacagggcctgcagctgttcttcagaaagttcaagaagatcaacttcgacaaggtggccagcaacttcgagttcgtgttcgactacaacgacttcaccaacaa
ggccgagggcaccaaaaccaactggaccatcagcacccagggaaccagaatcgccaagtacagaagcgatgacgccaacggcaagtggatcagcagaacc
gttcaccccaccgacattatcaaggaggccctgaacagagagaagatcaactacaacgacggccacgacctgatcgatgagatcgtgagcatcgagaagagcg
ccgtgctgaaggagatctactacggcttcaagctgaccctgcagctgagaaatagcaccctggccaatgaagaggagcaggaggactacatcatcagccccgtg
aagaacagcagcggcaactacttcgacagcagaatcaccagcaaggagctgccctgtgatgctgatgctaacggcgcctacaacatcgccagaaagggactgt
gggccctggaacagatcagaaacagcgagaacgtgagcaaggtgaagctggccatcagcaacaaggagtggttcgagtacacccagaacaacatccccagcc
tgtga

>HkCas12a (codon-optimised DNA sequence)
atgttcgagaagctgagcaacatcgtgagcatcagcaagaccatcagattcaagctgatccccgtgggcaaaaccctggagaacatcgagaagctgggcaagct
ggagaaggacttcgagagaagcgacttctaccccatcctgaagaacatcagcgacgactactacagacagtacatcaaggagaagctgagcgacctgaacctg
gactggcagaagctgtacgatgcccacgagctgctggatagcagcaagaaggagagccagaagaacctggagatgatccaggcccagtacagaaaggtgctg
ttcaacatcctgagcggcgagctggataaaagcggcgagaagaacagcaaggacctgatcaagaacaacaaggccctgtacggcaagctgttcaagaagcagt
tcatcctggaggtgctgcccgacttcgtgaacaacaacgacagctacagcgaggaggacctggaaggactgaacctgtacagcaagttcaccaccagactgaag
aacttctgggagaccagaaagaacgtgttcaccgacaaggacatcgtgaccgccattcccttcagagccgtgaacgagaacttcggcttctactacgacaacatca
agatcttcaacaagaacatcgagtacctggagaacaagatccccaacctggagaacgaactgaaggaggccgatatcctggacgacaacagaagcgtgaagga
ctacttcacccccaacggcttcaactacgtgatcacccaggacggcatcgatgtgtaccaggccatcagaggcggattcaccaaggagaacggcgaaaaggtgc
agggcatcaacgagatcctgaacctgacccagcagcagctgagaagaaagcccgagaccaagaacgttaagctgggcgtgctgaccaagctgagaaagcag

atcctggagtacagcgagagcaccagcttcctgatcgaccagatcgaggacgacaacgacctggtggacagaatcaacaagttcaacgtgagcttcttcgagag
cacagaggttagccccagcctgtttgagcagatcgagagactgtacaacgccctgaagagcatcaagaaggaggaggtgtacatcgacgccagaaacacccag
aagttcagccagatgctgttcggacagtgggacgtgatcagaagaggctacaccgtgaagattaccgagggcagcaaggaggagaagaagaagtacaaggag
tacctggagctggacgagacaagcaaggccaagagatacctgaacatcagagagatcgaggagctggtgaaccttgtggagggcttcgaagaggtggacgtgt
tcagcgtgctgctggagaagttcaagatgaacaacatcgagagaagcgagttcgaggcccctatctacggaagccctatcaagctggaggccatcaaggagtac
ctggagaagcacctggaggagtaccacaagtggaagctgctgctgatcggcaacgacgatctggataccgacgagaccttctacccccctgctgaacgaggtgat
cagcgactactacatcatccccctgtacaacctgaccagaaactacctgaccagaaagcacagcgacaaggacaagatcaaggtgaacttcgacttccccacact
ggctgacggatggagcgaaagcaagatcagcgacaacagaagcatcatcctgagaaagggcggctactactacctgggcatcctgatcgacaacaagctgctg
atcaacaagaagaacaagagcaagaagatctacgagatcctgatctacaaccagatccccgagttcagcaagagcatccccaactaccccttcaccaagaaggt
gaaggagcacttcaagaacaacgtgagcgacttccagctgatcgacggatatgtgagcccccctgatcatcaccaaggagatctacgacatcaagaaggagaaga
agtacaagaaggacttctacaaggacaacaacaccaacaagaactacctgtacaccatctacaagtggatcgagttctgcaagcagttcctgtacaagtacaaggg
ccccaacaaggagagctacaaggagatgtacgacttcagcaccctgaaggacaccagcctgtacgtgaacctgaacgacttctacgccgacgtgaacagctgtg
cctacagagtgctgttcaacaagatcgacgagaacaccatcgacaacgccgtggaggatggaaagctgctgctgttccagatctacaacaaggacttcagccccg
agagcaagggcaagaagaacctgcacaccctgtactggctgagcatgttcagcgaggagaacctgagaaccagaaagctgaagctgaacggccaggccgag
atcttctacagaaagaagctggagaagaagcccatcatccacaaggagggcagcatcctgctgaacaagatcgacaaggagggcaacaccatccccgagaac
atctaccacgagtgctacagatacctgaacaagaagatcggcagagaggacctgagcgacgaagctatcgccctgttcaacaaggacgtgctgaagtacaagga
ggccagattcgacatcatcaaggacagaagatacagcgagagccagttcttcttccacgtgcccatcaccttcaactgggacatcaagaccaacaagaacgtgaa
ccagatcgtgcagggcatgatcaaggacggcgagatcaagcacatcatcggcatcgacagaggcgaaagacacctgctgtactacagcgtgatcgacctggag
ggcaacatcgttgaacagggcagcctgaacacactggagcagaacagattcgacaacagcaccgtgaaggtggactaccagaacaagctgagaaccagagag
gaggacagagacagagccagaaagaactggaccaacatcaacaagatcaaggagctgaaggacggctatctgagccacgtggtgcacaagctgagcagact
gatcatcaagtacgaggccatcgtgatcatggagaacctgaaccagggcttcaagagaggcagattcaaggtggagagacaggtgtaccagaagttcgagctgg
ccctgatgaacaaactgagcgccctgagcttcaaggagaagtacgacgagagaaagaacctggagcccagcggaatcctgaatcccatccaggcctgttatccc
gtggacgcttaccaagaactgcagggacagaacggcatcgtgttctatctgcccgccgcttacacaagcgtgattgaccccgtgaccggctttaccaacctgttca
gactgaagagcatcaacagcagcaagtacgaggagttcatcaagaagttcaagaacatctacttcgacaacgaggaggaggacttcaagttcatcttcaactacaa
ggacttcgccaaggccaacctggtgatcctgaacaacatcaagagcaaggactggaagatcagcaccagaggcgagagaatcagctacaacagcaagaagaa
ggagtacttctacgtgcagcccaccgagttcctgatcaacaagctgaaggagctgaacatcgactacgagaacatcgacatcatccccctgatcgacaacctgga
ggagaaggccaagagaaagatcctgaaggccctgttcgacaccttcaagtacagcgtgcagctgagaaactacgacttcgagaacgactacatcatcagcccca
ccgccgatgataacggcaactactacaacagcaacgagatcgacatcgacaagaccaacctgcccaacaacggagatgctaacggcgccttcaacatcgctag
aaagggcctgctgctgaaggacagaatcgtgaacagcaacgagagcaaggtggacctgaagatcaagaacgaggactggatcaacttcatcatcagctga

>OsCas12a (codon-optimised DNA sequence)
atggagaccgagatcctgaagtacgacttcttcgagagagagggcaagtacatgtactacgacggcctgaccaaacaatacgccctgagcaagaccatcagaaa
cgagctggtgcccatcggaaaaaccctggacaacatcaagaagaacagaatcctggaggccgacatcaagagaaagagcgactacgagcacgtgaagaagct
gatggacatgtaccacaagaagatcatcaacgaggccctggacaacttcaagctgagcgtgctggaagatgccgccgacatctacttcaacaagcagaacgacg
agagagacatcgacgccttcctgaagatccaggacaagctgagaaaggagatcgtggagcagctgaagggacacaccgactacagcaaggtgggcaacaag
gactttctgggcctgctgaaagctgctagcaccgaggaagacagaatcctgatcgagagcttcgacaacttctacacctacttcaccagctacaacaaggtgagaa
gcaacctgtacagcgccgaggataagagcagcaccgtggcctacagactgatcaacgagaacctgcccaagttcttcgacaacatcaaggcctacagaaccgtg
agaaacgccggagtgatcagcggagacatgagcatcgtggagcaggacgagctgtttgaggtggacaccttcaaccacaccctgacccagtatggcatcgaca
cctacaaccacatgatcggccagctgaacagcgccatcaacctgtacaaccagaagatgcatggcgccggcagctttaaaaagctgcccaagatgaaggagctg
tacaagcagctgctgaccgagagagaggaggagttcatcgaggagtacaccgacgacgaggtgctgattaccagcgtgcacaactacgtgagctacctgatcg
actacctgaacagcgacaaggtggagagcttcttcgacaccctgagaaagagcgacggcaaggaggtgttcatcaagaacgacgtgagcaagaccaccatgag
caacatcctgttcgacaactggagcaccatcgacgacctgatcaaccacgagtacgacagcgctcccgagaacgtgaagaagaccaaggacgacaagtacttc

gagaagagacagaaggacctgaagaagaacaagagctacagcctgagcaagatcgccgctctgtgcagagataccaccatcctggagaagtacatcagaaga
ctggtggacgacatcgagaagatctacaccagcaacaacgtgttcagcgacatcgtgctgagcaagcacgacagaagcaagaagctgagcaagaacaccaatg
ccgtgcaggccatcaagaacatgctggacagcatcaaggacttcgagcacgacgtgatgctgattaacggcagcggccaggagatcaagaagaacctgaacgt
gtacagcgaacaagaagccctggccggaatacttagacaggtggaccacatctacaacctgaccagaaactacctgaccaagaagcccttcagcaccgagaag
atcaagctgaacttcaacagacccaccttcctggatggctgggacaagaataaggaggaggccaacctgggaatcctgctgatcaaggacaacagatactacctg
ggcatcatgaacaccagcagcaacaaggccttcgtgaaccccccctaaggccatcagcaacgacatctacaagaaggtggactacaagttactgcccggccccaa
taagatgctgcccaaggtgttcttcgccaccaagaacatcgcttactacgcccccagcgaagaactgctgagcaagtacagaaagggcacccacaagaaggga
gacagcttcagcatcgacgactgcagaaacctgatcgacttcttcaagagcagcatcaacaagaacaccgactggagcaccttcggcttcaacttcagcgacacc
aacagctacaacgacatcagcgacttctacagagaggtggagaagcagggctacaagctgagcttcaccgacatcgacgcctgctacatcaaggacctggtgga
caacaacgagctgtacctgttccagatctacaacaaggacttcagcccctacagcaagggcaagctgaacctgcacaccctgtacttcaagatgctgttcgaccag
agaaacctggacaacgtggtgtacaagctgaacggagaggccgaggtgttctatagacccgccagcatcgaatctgacgagcagatcatccacaagagcggcc
agaacatcaagaacaagaaccagaagagaagcaactgcaagaagaccagcaccttcgactacgacatcgtgaaggacagaagatactgcaaggacaagttcat
gctgcacctgcccatcacagtgaacttcggcacaaacgagagcggcaagttcaacgagctggtgaacaacgccatcagagccgacaaggacgtgaacgtgatc
ggcatcgacagaggcgagagaaacctgctgtacgtggtggtggtggatccctgtggcaagatcatcgagcagatcagcctgaacaccatcgtggacaaggagt
acgacatcgagaccgactaccaccagctgctggacgaaaaggagggcagcagagacaaggccagaaaggactggaacaccatcgagaacatcaaggagct
gaaggagggctatctgagccaggtggtgaacatcatcgccaagctggtgctgaagtacgacgccatcatctgcctggaggacctgaacttcggcttcaagagag
gcagacagaaggtggagaagcaggtgtaccagaagttcgagaagatgctgatcgacaagatgaactacctggtgctggacaagagcagaaagcaggagagcc
ctcaaaaacctggaggagccctgaatgccctgcaactgacaagcgccttcaagagcttcaaggagctgggaaagcagaccggcatcatctattacgtgcccgcct
acctgaccagcaagatcgatcctaccaccggcttcgccaacctgttctacatcaagtacgagagcgtggacaaggccagagacttcttcagcaagttcgacttcatc
agatacaaccagatggacaactacttcgagttcggcttcgactacaagagcttcaccgagagagccagcggctgtaagagcaagtggatcgcctgtaccaacgg
cgagagaatcgtgaagtacagaaacagcgacaagaacaacagcttcgacgacaagaccgtgatcctgaccgacgagtacagaagcctgttcgacaagtacctg
cagaactacatcgacgaggacgacctgaaggaccagatcctgcagattgacagcgccgacttctacaagaacctgatcaagctgttccagctgaccctgcagatg
agaaacagcagcagcgacggcaagagagactacatcatcagccccgtgaagaactacagagaggagttcttctgcagcgagttcagcgatgacaccttcccca
gagacgctgatgctaacggcgcctacaacatcgctagaaagggcctgtgggtgatcaagcagatcagagagaccaagagcggcaccaagatcaacctggccat
gagcaactctgagtggctggagtatgcccagtgcaacctgttatga

>TsCas12a (codon-optimised DNA sequence)
atgaccaagaccttcgacagcgagttcttcaacctgtacagcctgcagaagaccgtgagattcgagctgaaacccgtgggagaaacagctagcttcgtggaggac
ttcaagaacgagggcctgaagagagtggtgagcgaggacgaaagaagagccgtggactaccagaaggtgaaggagatcatcgacgactaccacagagacttc
atcgaggagagcctgaactacttccccgagcaggtgagcaaagacgctctggagcaggcctttcacctgtaccagaagctgaaggccgctaaggtggaggaaa
gagagaaggccctgaaggaatgggaagccctgcagaagaagctgagagagaaggtggtgaagtgcttcagcgacagcaacaaggccagattcagcagaatc
gacaagaaggagctgatcaaggaggacctgatcaactggctggtggcccagaatagagaggacgacatccctaccgtggagaccttcaacaacttcaccaccta
cttcaccggcttccacgagaacagaaagaacatctacagcaaggacgaccacgctaccgccatcagcttcagactgatccacgagaacctgcccaagttcttcga
caacgtgatcagcttcaacaagctgaaggagggcttccccgagctgaagttcgacaaggtgaaggaggacctggaggtggactacgatctgaagcacgccttcg
agatcgagtacttcgtgaacttcgtgacccaggccggaatcgaccagtacaactacctgctgggcggcaaaacactggaggatggcacaaagaagcagggcat
gaacgagcagatcaacctgttcaagcagcagcagaccagagacaaggccagacagatccccaaactgatcccccctgttcaagcagatcctgagcgagagaacc
gagagccagagcttcatccccaagcagttcgagagcgaccaggagctgtttgacagcctgcagaagctgcacaacaactgccaggacaagttcaccgtgctgca
gcaggctattctgggactggctgaggctgatctgaagaaggtgttcatcaagaccagcgacctgaacgctctgagcaacaccatcttcggcaactacagcgtgttt
agcgacgccctgaacctgtacaaggagagcctgaagaccaagaaagcccaggaggccttcgaaaaactgcccgcccatagcatccatgacctgatccagtacc
tggagcagttcaacagcagcctggacgctgaaaagcagcagagcacagacaccgtgctgaactacttcatcaagaccgacgagctgtacagcagattcatcaag
agcaccagcgaggctttcacccaagtgcaacccctgtttgagctggaagccctgagcagcaaaagaagaccccccgagagcgaagatgaaggcgctaagggc
caagaaggcttcgagcagatcaagagaatcaaggcctacctggacacacttatggaggccgtgcactttgctaagcccctgtatctggtgaagggcagaaagatg

atcgagggcctggacaaggaccagagcttctacgaggccttcgagatggcctaccaggaactggagagcctgatcatccccatctacaacaaggccagaagcta
cctgagcagaaagcccttcaaggccgacaagttcaagatcaacttcgacaacaacaccctgctgtctggatgggacgccaacaaggaaacagccaacgccagc
atcctgttcaagaaggacggcctgtactaccttggcatcatgcccaagggcaagaccttcctgttcgactacttcgtgagcagcgaggacagcgagaagctgaag
cagagaagacagaagaccgccgaggaagctcttgctcaagacggcgaaagctacttcgagaagatcagatacaagctgctgcccggcgctagcaaaatgctgc
ccaaggtgttcttcagcaacaagaacatcggcttctacaaccccagcgacgacatcctgagaatcagaaacaccgccagccacaccaaaaatggcacccccag
aagggacatagcaaggtggagttcaacctgaacgactgccacaagatgatcgacttcttcaagagcagcatccagaagcaccccgaatggggaagcttcggctt
cacattcagcgacaccagcgacttcgaagacatgagcgccttctacagagaggtggagaaccagggctacgtgatcagcttcgacaagatcaaggagacctaca
tccagagccaggtggagcagggaaacctgtacctgttccagatctacaacaaggacttcagcccctactctaagggcaagcccaatctgcacaccctgtactgga
aggccctgttcgaagaggccaacctgaataacgtggtggccaagctgaatggcgaagccgagatcttcttcagaagacacagcatcaaggccagcgacaaggt
ggtgcaccctgccaatcaagccatcgacaacaagaacccccacaccgagaaaacccagagcaccttcgagtacgacctggtgaaggacaagagatacaccca
ggacaagttcttcttccacgtgcccatcagcctgaactttaaggcccagggcgtgagcaagttcaacgacaaggtgaacggcttcctgaagggaaaccccgacgt
gaacatcatcggcatcgacagaggcgaaagacacctgctgtacttcaccgtggtgaaccagaagggcgagatccttgtgcaggagagcctgaacaccctgatga
gcgacaagggccacgtgaacgactaccagcagaagctggacaagaaggagcaggaaagagacgccgccagaaaatcttggaccaccgtggagaacatcaa
ggagctgaaggagggctatctgagccacgtggtgcataagctggcccacctgatcatcaagtacaacgccatcgtgtgcctggaggacctgaacttcggcttcaa
gagaggcagattcaaggtggagaagcaggtgtaccagaagttcgagaaggccctgatcgacaagctgaactacctggtgttcaaggagaaggagctgggcga
agtgggacattatctgaccgcctaccaactgacagctcccttcgagagcttcaagaagctgggcaagcagagcggcatcctgttttacgtgcccgctgactacacc
tctaagatcgaccccacaaccggcttcgtgaacttcctggacctgagataccagagcgtggagaaggctaagcagctgctgagcgacttcaacgccatcagattc
aacagcgtgcagaactacttcgagttcgagatcgactacaagaagctgacccccaagagaaaagtgggcacccagagcaaatgggtgatctgcacctacggcg
acgtgagataccagaacagaagaaaccagaagggccactgggaaaccgaagaggtgaacgtgaccgagaagctgaaggctctgttcgccagcgacagcaag
acaaccaccgtgatcgactacgccaacgacgacaacctgatcgacgtgatcctggagcaagacaaggccagcttcttcaaggagctgctgtggctgctgaagct
gaccatgaccctgagacacagcaagatcaagagcgaggacgacttcattctgagccccgtgaagaatgagcagggcgagttctacgacagcagaaaggccgg
agaagtgtggcccaaagatgccgatgctaacggagcctaccatatcgctctgaagggcctgtggaacctgcagcagatcaaccagtgggagaagggcaagaca
ctgaacctggccatcaagaaccaggactggttcagcttcatccaggagaagccctaccaggaatga

>BbCas12a (codon-optimised DNA sequence)
atgaagaagttcaccaacctgtaccccgtgcagaagaccctgagattcgagcttatcccccagggcaatacctctaagcacctgtgcaagatcatccaggaggac
gagcagatcgctgaggatagccaggaggtgaagaagctgctggacagataccacaaggagttcatcgccatcgccctgagcagctttcctacaagccccctggc
caaagagatcatccccaagctgaaggagttcgcccagatcagagctacaggcgacgccaagcagattagcaccatccaggacgagctgagagagcttgtggtg
aagggctttaagggagagggcgagcaggagagaagatacaagatcctgatcggcgctaagggcaaccctaatgctgacgagctgttcaacaccgagctgatca
acttcctgaaggaccctgctgaacaggctctggtgaagaagttccagaagcacaccggctacttcctgggcttcaacgagaacagaaagaacatgtacagcgcca
aggctcagagcacagccattgcctacagactgatccacgagaacctgcccagattcctggacaacatcaccacctacgagaaggtgaagacctacctgaaggag
gagatcccccagctggagaaggaactggtgagagccggagcttctctggttagccatgtggacagcgtgttcaccatcgacttcttcctggaggtgttcacccaaa
gcggcatcgaccaatacaacgccctgatcggcaagatcgtgaaccaagagcagggcgaggtgaaaggcctgaacgagagaatcaacctgtacaaccagcagc
acaagcaggaggctaagctgcctctgttcaagcccctgtacaagcagatcctgagcgacagagagcaacttagctggctggccgaagcttacaacagcgacaag
gacctgctggacagcatccagaagtactaccagctgctgatcgacaaccagatcttcgagagaatccccagactgatgcacacactggagaaagccccccctgga
caagatctggatcacctacgacacccagctgaccagcatcagcaacaccctgtacggcagctggagagtgattggagaggccctgggcagaaatgccaagagc
gagaaggagagaaagagcagccagaagaaggccctgaactacagcctggagagcatcaatcaggccatcgccaagatgcccagcgatgaggaacttcctccc
atccagaagtacttcatcgccctgggaagcaaccccagcaagaaagacgctagcagcggcacactggtggataaggtgagaagcagctacaaggcctgccag
gacatccttaccaaccctgaccataccggcaagaagctgatccaggacaagaagcaggtggacctgctgaaacagctgctggacgaccttctgatcctgcagag
attcatcaagcccctgctgtacagcaacaacgagaacgagacccacaaggacgaggtgttctacaccgagctgaccgacatcatggacctgctgaaccctatcgt
gggcctgtacaacaaggtgagaaactacctgacccagaagccctacagcaccgagaagttcaagatcaacttcaagagcagcagcctgctggctggatgggac
agaaacaaggagaaggacaacctgggcgtgatcctgaagagagaggacaagtactacctggccatcatggacaaggctcacaacgccaccttcaagaacaag

agcctgcccacacaaggagagtgctacgagaagatggagtacaagctgctgcccggcgccaataaaatgctgcccaaggtgtacatcaccagcaagaagggc
atcgagagcttccatcccagcgaagagctgcagaagaagtacaagctgggcacccacaagaaaggagccagcttcaacctgagcgacatgagagccctgatc
gactacttcaaggagagcctggagaagcatgaggagcacagccaattcggcttccacttcagcgacaccagcacctacgaagacatcagcggcttctacagaga
ggttgagcagcaggcctacaagatcaccttcagaaaggtgagcgtggagtacatcgaccagctggtgaacgagggcaagctgtacctgttccagatctacaaca
aggacttcagcccctacagcaagggcacccctaatctgcacaccctgtactggaagatgctgttcgaccccgccaatctgcaggacatcgtgtacaagctgaacg
gagaggccgaggtgttcttcagaaagaagagcctgcagtacgacagacccacacaccctaagggccaacccatcaacaagaagagcctgctgaacgaaggag
agaccagcctgtttgactacgacctgatcaaggacagaagattcaccgtggacaagttccagttccacgtgcccatcaccatgaacttcaaggccacccagggca
ccaaagtgaaccagatggtgcaggaggaggtgaagaagagcaagggcttccacctgatcggaatcgacagaggcgagagaaacctgctgtacatcgtggtgat
caacgagagaggcgagatcatcgagcagtgcagcctgaacaagatcgtgaacacctaccaggagaaggagcacaccgtggactataaggccctgctggagaa
gagaagccagagcagactggaggagagaaagagctggcagaccatcgagaacatcaaggagctgaagggcggctacctgtctcaagtggtgcacaaaatcg
cccagctgatgatcaagtacaacgccatcgccgtgctggaggatctgaacttcggcttcatcagaaccagaaagaagttcgagttcagcgtgtaccaggagttcga
gaagaagctgatcgacaagctgggctacgtggtggataaaaaggcccccatccaacaagaaggaggactgctgcaggcttatcaactgacagccccccttcaaga
gcttcagagagatgggcaagcagaacggcttcctgttctatgtgcccgcctggaatacctctgccatcgaccctagaacaggcttcgtgaacctgctggacaccag
atacgagagcatcgccaagaccaaggagctgatcaagaagctgaaggacatcagatacaacagccagaaggactggttcgagatcgacctggactacaacgcc
ttcggcaacagagctaagggcagcagaagcaagtggagactgtgcagctacggcgagagaatcgagcacaccagaaagcaggacagcaacggccaggaag
aaagcgacagcatggtggttctgaccgaggccttcaaggacgtgttcaccaagtaccagatcgactacagagagaacctgaaggagcagctgctgctgcaaagc
gacaaggccttcttcgtggacttcctgagcctgctgagactgaccctgcagctgagaaacagcctgagcaacagcctgatcgactacatcctgagccccgtggct
gatgaaaacggcgagttcttcgacagcagaaaggccctgagcaacgaacctcaagatgccgacgccaacggagcttatcacatcgccctgaaaggactgtggg
tgctggacaagatcagaaagaccgagaaggtgacccctgccaaactggctctgagcaaccaggaatggctgagcttcgctcaggagaagcccttcttcaacgag
tga

>BoCas12a (codon-optimised DNA sequence)
atgaggaagttcaacgagttcgtgggcctgtaccccattagcaagaccctgagattcgagctgaagcccatcggcaaaaccctggagcacatccagagaaacaa
gctgctggagcacgatgctgtgagagctgacgattacgtgaaggtgaagaagatcatcgacaagtaccacaagtgcctgatcgatgaggctctgagcggcttcac
atttgacacagaggccgacggcagaagcaataacagcctgagcgagtactacctgtactacaacctgaagaagagaaacgagcaggagcagaagaccttcaag
accatccagaacaacctgagaaagcagatcgtgaacaagctgacccagagcgagaagtacaagagaatcgacaagaaggagctgatcaccaccgatctgccc
gacttcctgacaaacgagagcgagaaggagctggtggagaagttcaagaacttcaccacctacttcaccgagttccacaagaacagaaagaacatgtacagcaa
ggaggagaagagcaccgccatcgccttcagactgatcaacgagaacctgcccaagttcgtggataacatcgccgccttcgagaaagttgtgagcagccccctgg
ccgaaaaaatcaacgccctgtacgaggacttcaaggagtacctgaacgtggaggagatcagcagagtgttcagactggactactacgacgagctgctgacccag
aagcagatcgacctgtacaacgccattgtgggcggcagaaccgaagaggacaacaagatccagatcaagggcctgaaccagtacatcaacgagtacaaccag
cagcagaccgacagaagcaacagactgcccaagctgaagcccctgtacaagcagatcctgagcgacagagagagcgttagctggctgccccctaaattcgaca
gcgacaagaacctgctgatcaagatcaaggagtgctacgacgcccttagcgagaaggagaaggtgttcgacaagctggagagcatcctgaagagcctgagcac
ctacgacctgagcaagatctacatcagcaacgacagccagctgagctacatcagccagaagatgttcggcagatgggacatcatcagcaaggccatcagagag
gactgcgccaagagaaacccccagaagagcagagagagcctggagaagttcgccgagagaatcgacaagaagctgaagaccatcgacagcatcagcatcgg
cgacgtggacgaatgtcttgctcagctgggcgaaacctacgtgaagagagtggaggactacttcgtggctatgggcgagagcgaaattgacgacgagcagacc
gataccaccagcttcaagaagaacatcgagggcgcctacgaatctgtgaaggagctgctgaacaacgccgacaacatcaccgacaacaacctgatgcaggaca
agggcaacgtggagaagatcaagaccctgctggacgccattaaggacctgcagagattcatcaagcccctgctgggcaaaggagatgaggccgataaagacg
gcgtgttctacggcgaatttaccagcctgtggaccaaactggaccaagtgaccccccctgtacaacatggtgagaaactacctgaccagcaagccctacagcacca
agaagatcaagctgaacttcgagaacagcaccctgatggatggctgggacctgaacaaagagcccgacaacaccaccgtgatcttctgcaaggacggcctgtac
taccttggcatcatgggcaagaagtacaacagagtgttcgtggacagagaggatctgcctcacgacggagagtgctacgacaagatggagtacaagctgcttccc
ggcgccaataaaatgctgcccaaggtgttctttagcgagaccggcatccaaagattcctgcccagcgaagaactgctgggcaagtacgaaagaggcacccacaa
gaagggagccggatttgacctgggagactgcagagctctgatcgacttcttcaagaagagcatcgagagacacgacgactggaagaagttcgacttcaagttcag

cgacaccagcacctaccaggacatcagcgagttctacagagaggtggagcagcagggctacaagatgagcttcagaaaggtgagcgtggactacatcaagag
cctggtggaggagggaaagctgtacctgttccagatctacaacaaggacttcagcgcccactctaagggaacacccaacatgcacaccctgtactggaagatgct
gttcgacgaggagaacctgaaggacgtggtgtacaagctgaacggagaggccgaggtgttcttcagaaagagcagcatcaccgtgcagagccctacacatcct
gccaacagccccatcaagaacaagaacaaggacaaccagaagaaggagagcaagttcgagtacgacctgatcaaggacagaagatacaccgtggacaagttc
ctgttccacgtgcccatcaccatgaacttcaagagcgtgggcggcagcaacattaaccagctggtgaagagacacatcagaagcgccaccgacctgcacattatc
ggcatcgacagaggcgaaagacacctgctgtacctgaccgtgatcgacagcagaggcaacatcaaggagcagttcagcctgaacgagatcgtgaacgagtaca
acggcaacacctacagaaccgactaccacgagctgctggacacaagagagggcgaaagaaccgaggccagaagaaactggcagaccatccagaacatcag
agagctgaaggagggctatctgagccaggtgatccacaagatcagcgagctggccatcaagtacaacgccgtgatcgtgctggaggacctgaacttcggcttcat
gagaagcagacagaaggtggagaagcaggtgtaccagaagttcgagaagatgctgatcgacaagctgaactacctggtggacaagaagaagcccgtggctga
aacaggaggactgctgagagcctatcaactgaccggcgagttcgagagctttaagaccctgggcaagcaaagcggcatcctgttctacgtgcccgcttggaaca
ccagcaagatcgatcccgtgaccggctttgtgaacctgttcgacacccactacgagaacatcgagaaggccaaggtgttcttcgacaagttcaagagcatcagata
caacagcgacaaggactggttcgagttcgtggtggacgactacaccagattcagccccaaggccgaaggaaccagaagagactggaccatctgcacccaggg
caagagaatccagatctgcagaaaccaccagagaaacaacgagtgggagggccaagaaatcgacctgaccaaggccttcaaggagcacttcgaggcctatgg
cgtggacatcagcaaggacctgagagagcagatcaacacccagaacaagaaggagttcttcgaggagctgctgagactgctgagactgaccctgcagatgaga
aacagcatgcccagcagcgacatcgactacctgatcagccccgtggctaatgacacaggctgcttcttcgacagcagaaagcaggccgagctgaaggaaaatg
ccgtgctgcctatgaatgccgatgccaacggagcttacaacatcgccagaaagggcctgctggccatcagaaagatgaagcaggaggagaacgacagcgcca
aaatcagcctggccatcagcaacaaggagtggctgaagttcgctcagaccaagccctatctggaggactga

>Lb4Cas12a (codon-optimised DNA sequence)
atgggactgtatgacggcttcgtgaacagatacagcgtgagcaagaccctgagattcgagctgatcccccagggaagaaccagagagtacatcgagaccaacg
gcatcctgagcgacgatgaggagagagccaaggactacaagaccatcaagagactgatcgacgagtaccacaaggactacatcagcagatgcctgaagaacg
tgaacatcagctgcctggaggagtactaccacctgtacaacagcagcaacagagacaagagacacgaggagctggatgctctgagcgaccaaatgagaggcg
agatcgccagctttctgaccggcaacgacgagtacaaggagcagaagagcagagacatcatcatcaacgagagaatcatcaacttcgccagcaccgatgaaga
actggccgccgtgaagagattcagaaagttcaccagctacttcaccggcttcttcaccaacagagagaacatgtacagcgccgagaagaagtctacagccatcgc
ccacagaatcatcgacgtgaacctgcccaagtacgtggacaacatcaaggccttcaacaccgctattgaagccggcgtgttcgatattgccgagttcgagagcaa
cttcaaggccatcaccgacgaacatgaggtgagcgacctgctggacatcaccaagtacagcagattcatcagaaacgaggacatcatcatctacaacaccctgct
gggcggcattagcatgaaggacgagaagatccagggcctgaacgagctgatcaacctgcacaaccagaagcaccccggcaaaaaggtgcctctgctgaaggt
gctgtacaagcagatcctgggcgacagccagacacatagcttcgtggacgaccagttcgaggatgaccagcaggtgatcaacgccgtgaaggctgtgaccgac
acctttagcgagaccctgctgggaagcctgaagatcatcatcaacaacatcggccactacgacctggacagaatctacatcaaggccggccaggacattacaacc
ctgagcaagagagccctgaacgactggcacatcatcaccgagtgcctggagtctgagtacgacgacaagttccccaagaacaagaagagcgacacctacgag
gagatgagaaacagatacgtgaagagcttcaagagcttcagcatcggcagactgaacagcctggtgaccacctataccgagcaagcctgcttcctggagaattac
ctgggcagcttcggaggcgataccgacaagaactgcctgaccgacttcaccaacagcctgatggaggtggagcacctgctgaacagcgaataccccgtgacca
acagactgatcaccgactacgagagcgtgagaatcctgaagagactgctggacagcgagatggaggtgatccacttcctgaaacccctgctgggcaatggaaac
gagagcgacaaggacctggtgttctacggcgagttcgaggccgagtacgaaaaactgctgcccgtgatcaaggtgtacaacagagtgagaaactacctgacca
gaaagcccttcagcaccgagaagatcaagctgaacttcaacagccccacactgctgtgtggatggagccagagcaaggagaaggagtacatgggcgtgatcct
gagaaaggacggccagtactacctgggaatcatgacccccagcaacaagaagatcttcagcgaggctcccaagcctgatgaagactgctacgagaagatggtg
ctgagatacatcccccacccctatcagatgctgcccaaggtgttcttcagcaagagcaacatcgccttcttcaaccccagcgacgagatcctgagaatcaagaagc
aggagagcttcaagaagggcaagagcttcaacagagacgactgccacaagttcatcgacttctacaaggacagcatcaacagacacgaggagtggagaaagtt
caacttcaagttcagcgacaccgacagctacgaggacatcagcagattctacaaggaggtggagaaccaggccttcagcatgagcttcaccaagatccccaccg
tgtacatcgatagcctggtggacgagggaaagctgtacctgttcaagctgcacaacaaggacttcagcgagcacagcaagggcaaacccaacctgcacaccgtt
tactggaacgccctgttcagcgagtacaacctgcagaacaccgtgtaccagctgaacggaagcgccgagatcttcttcagaaaggccagcatccccgagaacga
gagagtgatccacaagaagaacgtgcccatcaccagaaaagtggccgagctgaacggcaaaaaggaggtgagcgtgttcccctacgacatcatcaagaacag

aagatacaccgtggacaagttccagttccacgtgcccctgaagatgaacttcaaggccgacgagaagaagagaatcaacgacgacgtgatcgaggccatcaga
agcaacaagggcatccacgtgatcggaatcgacagaggcgagagaaacctgctgtacctgagcctgatcaacgaggagggcagaatcatcgagcagagaagc
ctgaacatcatcgacagcggcgaaggccatacccagaactacagagacctgctggacagcagagagaaggacagagagaaggccagagagaactggcagg
agatccaggagatcaaggacctgaagaccggctatctgagccaagccatccacaccatcaccaagtggatgaaggagtacaacgccatcatcgtgctggagga
cctgaacgacagattcaccaacggcagaaagaaggtggagaagcaggtgtaccagaagttcgagaagatgctgatcgacaagctgaactactacgtggacaag
gacgaggagttcgacagaatgggcggcacacatagagctctgcaactgaccgagaagttcgagagcttccagaagctgggaagacagaccggcttcatcttcta
tgtgcccgcctggaacacatctaagctggatcccacaaccggattcgtggacctgctgtaccccaagtacaagagcgtggacgccaccaaggacttcatcaagaa
gttcgacttcatcagattcaacagcgagaagaactacttcgagttcggcctgcactacagcaacttcaccgagagagccatcggatgcagagacgagtggatcctg
tgcagctacggcaacagaatcgtgaacttcagaaacgccgccaagaacaacagctgggactacaaggagatcgacatcaccaagcagctgctggacctgttcg
agaagaacggcatcgacgtgaagcaggagaacctgatcgacagcatctgcgagatgaaggacaagcccttcttcaagagcctgatcgccaacatcaagctgatc
ctgcagatcagaaacagcgctagcggcaccgacatcgactacatgatcagccccgccatgaatgacagaggcgagttcttcgacaccagaaagggcctgcaac
aactgcctctggacgctgatgctaacggcgcctataacatcgccaagaagggcctgtggatcgtggaccagatcagaaacaccaccggcaacaacgtgaagatg
gccatgagcaacagagagtggatgcacttcgcccaggaaagcagacttgcttga

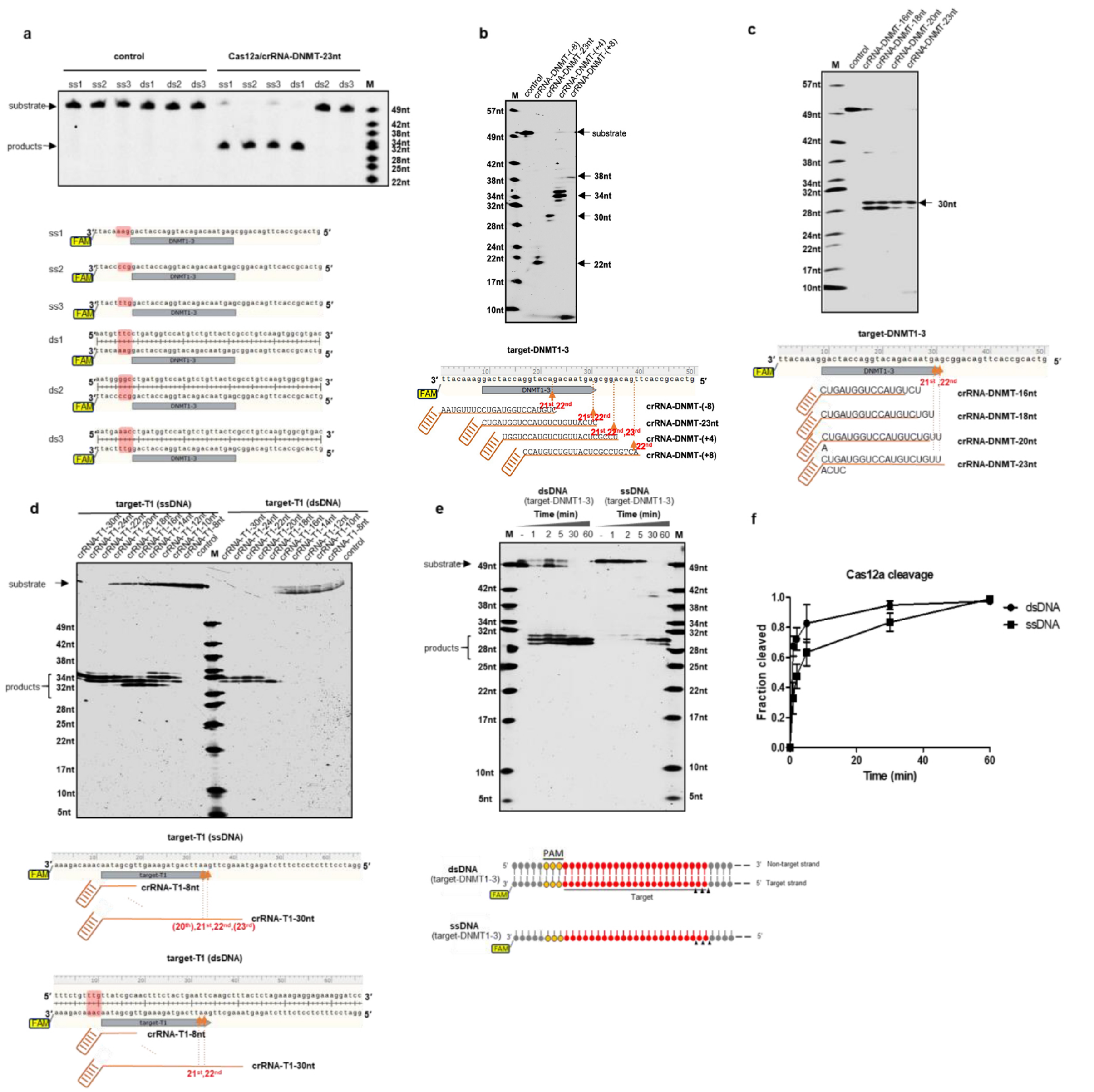

Figure S1 Cleavage of ssDNA by Cas12a with a programmable and PAM-independent mechanism. **(a)** Cleavage of ssDNA and dsDNA by Cas12a. 3′-FAM-labelled ssDNA or dsDNA containing different PAMs were cleaved by Cas12a and crRNA-DNMT-23nt. For dsDNA, only ds1 with the PAM sequence of "TTC" was successfully cleaved; however, for ssDNAs, all targets with or without PAM sequences could be cleaved. Schematic of the substrates was shown in the lower panel. **(b)** Identification of the Cas12a cleavage sites on ssDNA of target-DNMT1-3 using urea PAGE. The cleavage sites were near the 22nd base downstream of the first crRNA-paired base with different crRNAs. Schematic of the ssDNA substrate and crRNAs was shown in the lower panel. Paired guide sequences were shown by orange lines and the cleavage sites were indicated by both arrows and numbers. **(c)** Determination of the cleavage sites of Cas12a on target-DNMT1-3 ssDNA. crRNAs had short guide sequences of 16 nts, 18 nts and 20 nts, respectively. As there were 8 nts before the paired site in the target ssDNA, the 30-nt product indicated the cleavage of target ssDNA after the 22nd base by Cas12a. Schematic of the ssDNA substrate and crRNAs was shown in the lower panel. **(d)** crRNAs with different lengths of guide sequences were tested on dsDNA and ssDNA. At least 16-nt guide sequences were required by Cas12a for cleavage of target dsDNA;

however, even 10-nt guide sequence of crRNA enabled the cleavage of ssDNA by Cas12a. Schematic of the target DNA (target-T1) and the crRNAs was shown in the lower panel. **(e)** Time-course cleavage experiment. FAM was labelled on the 3′-end of the T-strand of dsDNA or target ssDNA, with the schematic of DNA substrates shown in the lower panel. Target sequence was shown in red and the PAM site of “TTC” was shown in yellow. **(f)** Quantification of the Cas12a DNA cleavage activity on dsDNA and ssDNA substrates. Cleavage assays were conducted in triplicate and data were represented as mean ± SEM.

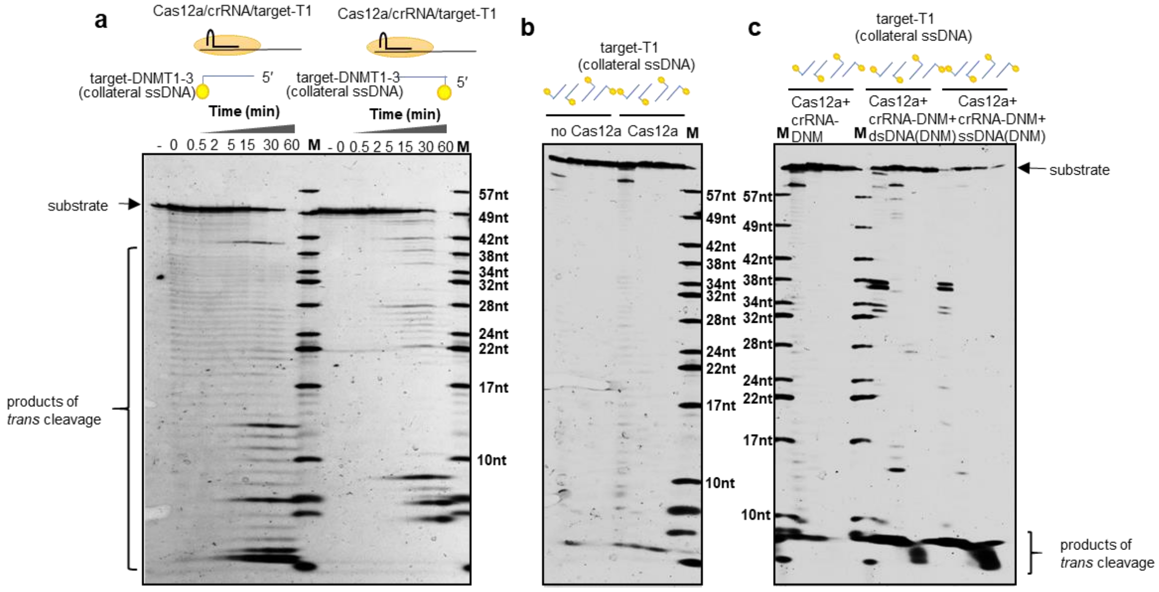

Figure S2 The Cas12a *trans* cleavage activity on ssDNA. **(a)** Time-course cleavage of DNMT1-3 ssDNA in the presence of Cas12a/crRNA-T1-24nt/target-T1 complex. Collateral ssDNAs (DNMT1-3) labelled with FAM at either the 3′-end (left part) or 5′-end (right part) were promiscuously cleaved. The concentration of crRNA and Cas12a used here was reduced to 100 nM each. **(b and c)** Cleavage of FAM-labelled collateral ssDNAs (i.e. target-T1-F-FAM, target-T1-R-FAM, target-T1-FAM-3′-F and target-T1-FAM-5′-R) by Cas12a **(b)** or its complex **(c)**. Digestion reactions with no Cas12a or only Cas12a were employed as negative controls **(b)**. When only Cas12a and crRNA-DNMT-23nt were added, collateral ssDNAs (target-T1, FAM-labelled) could not be cleaved. However, upon the formation of the ternary complex of Cas12a/crRNA-DNMT-23nt/target-DNMT1-3 (dsDNA or ssDNA) **(c)**, collateral ssDNAs (target-T1, FAM-labelled) were *trans* cleaved.

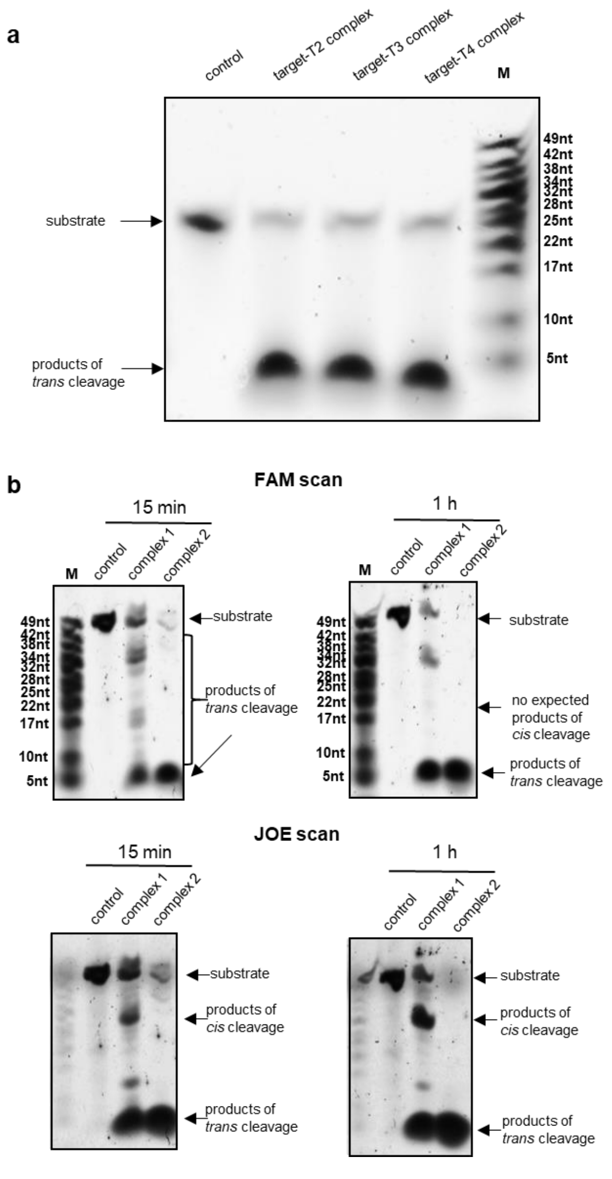

Figure S3 Cas12a-mediated *cis*- and *trans*-cleavage of ssDNA. **(a)** Collateral ssDNA substrate N25-3′FAM was *trans*-cleaved by the Cas12a complexes. All complexes, including Cas12a/crRNA-T2/target-T2, Cas12a/crRNA-T3/target-T3 and Cas12a/crRNA-T4/target-T4, had the ssDNA *trans*-cleavage activity. **(b)** Cleavage of double-end labelled ssDNA substrate (target-DNMT1-3-R-FAM-5′-JOE-3′), which was labelled with 5′-FAM and 3′-JOE. Double-end labelled ssDNA was cleaved for 15 min and 1 h by complex 1 (Cas12a/crRNA-DNMT-23nt/target-DNMT1-3-R-FAM-5′-JOE-3′) or complex 2 (Cas12a/crRNA-T1/target-T1-24-R). 3′-JOE-labelled *cis*-cleavage product (about 30 nt) only appeared with JOE scan, and no 5′-FAM-labelled *cis*-cleavage product (expected at 20 nt) could be observed.

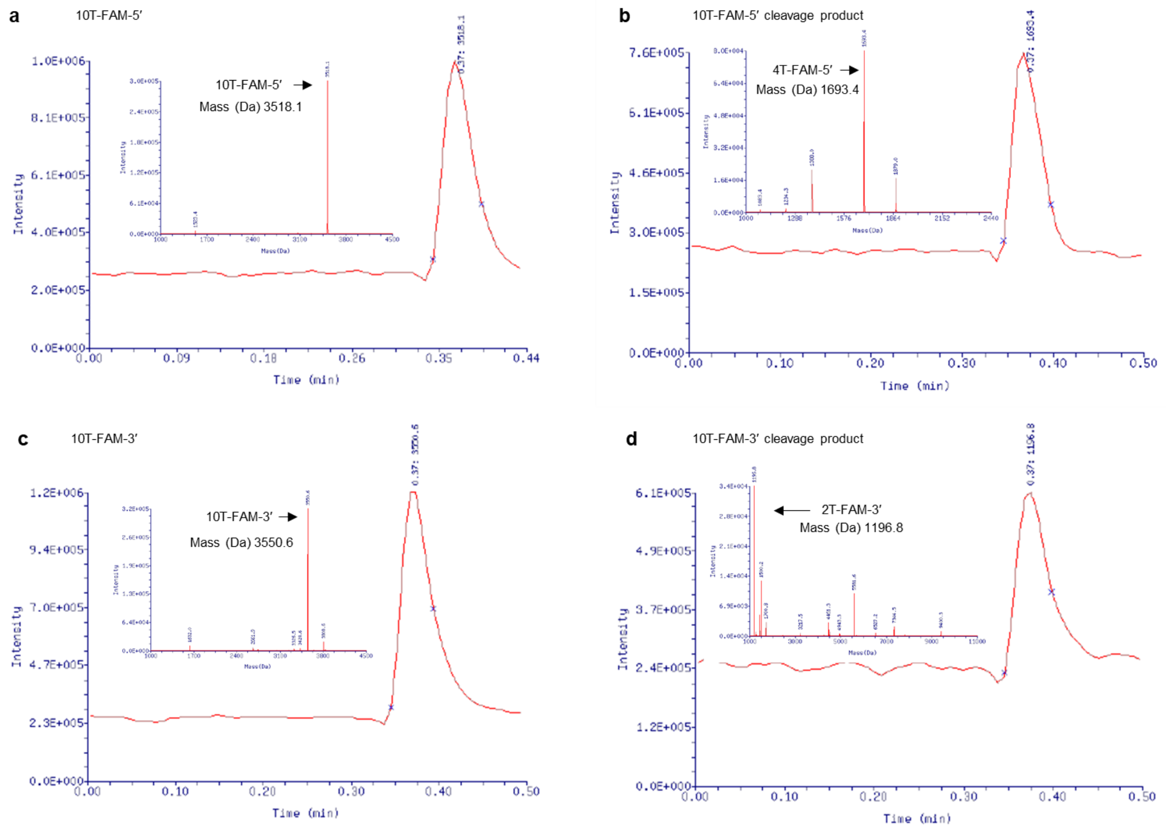

Figure S4 Analysis of ssDNA by LC-MS. Analysed ssDNAs included 10T-FAM-5′ **(a)**, 10T-FAM-3′ **(c)** and their corresponding products **(b and d)** *trans*-cleaved by complex of Cas12a/crRNA-T1/target-T1-18-R. The main cleavage product of substrate 10T-FAM-5′ was 4T-FAM-5′ with the molecular weight of 1693.4 **(b)**, and main cleavage product of 10T-FAM-3′ was 2T-FAM-3′ with the molecular weight of 1196.8 **(d)**.

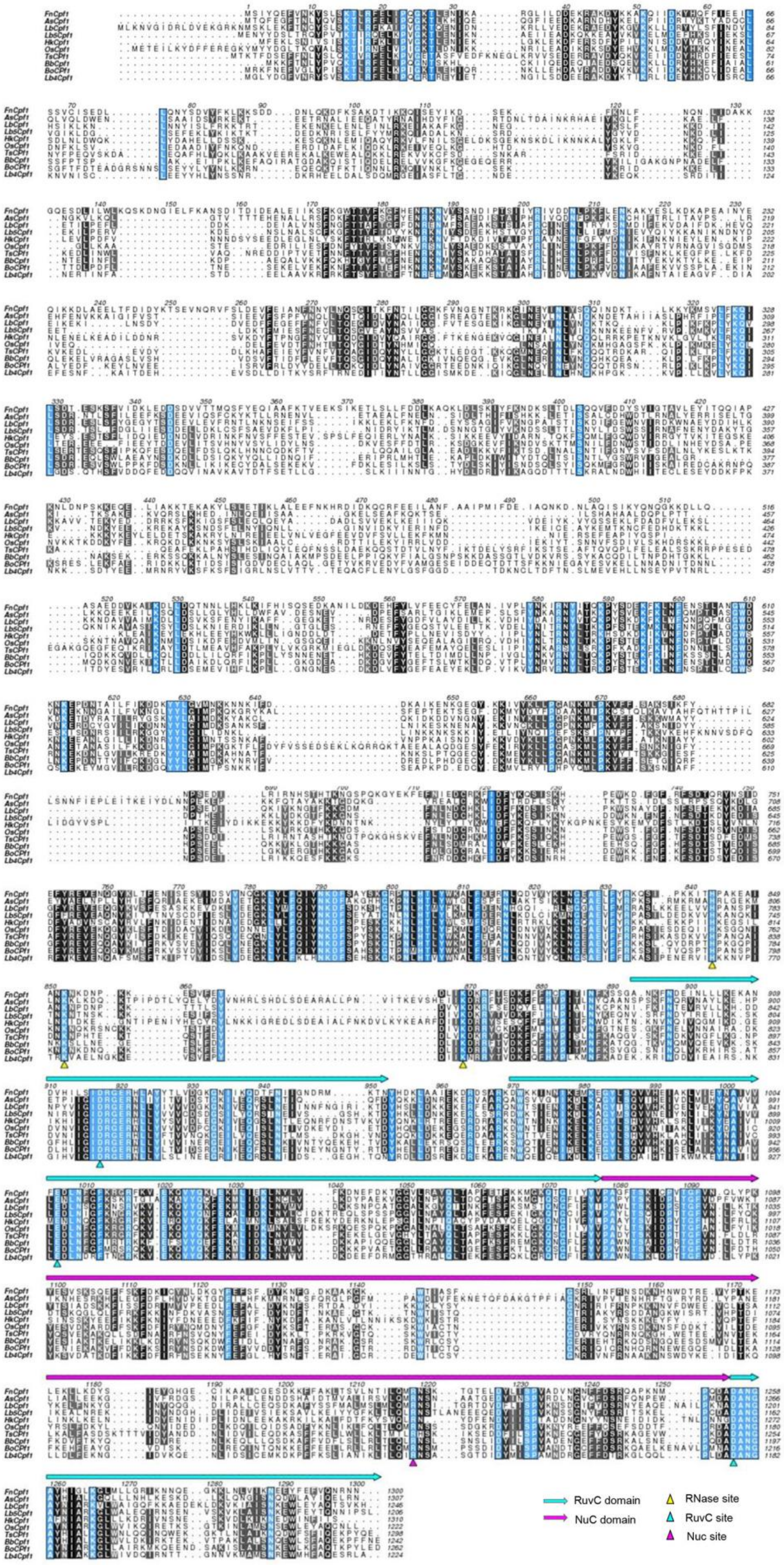

RuvC domain
NuC domain
RNase site
RuvC site
Nuc site

Figure S5 Multiple sequence alignment of Cas12a proteins. The RuvC and Nuc domains were labelled above the amino acid sequences, and key residues were indicated by coloured triangles. Fn, *Francisella tularensis;* As, *Acidaminococcus sp*. BV3L6; Lb, *Lachnospiraceae bacterium* ND2006; Lb5, *Lachnospiraceae bacterium* NC2008; Hk, *Helcococcus kunzii* ATCC 51366; Os, *Oribacterium sp.* NK2B42; Ts, *Thiomicrospira sp.* XS5; Bb, *Bacteroidales bacterium* KA00251; Bo, *Bacteroidetes oral taxon* 274 str. F0058; Lb4, *Lachnospiraceae bacterium* MC2017.

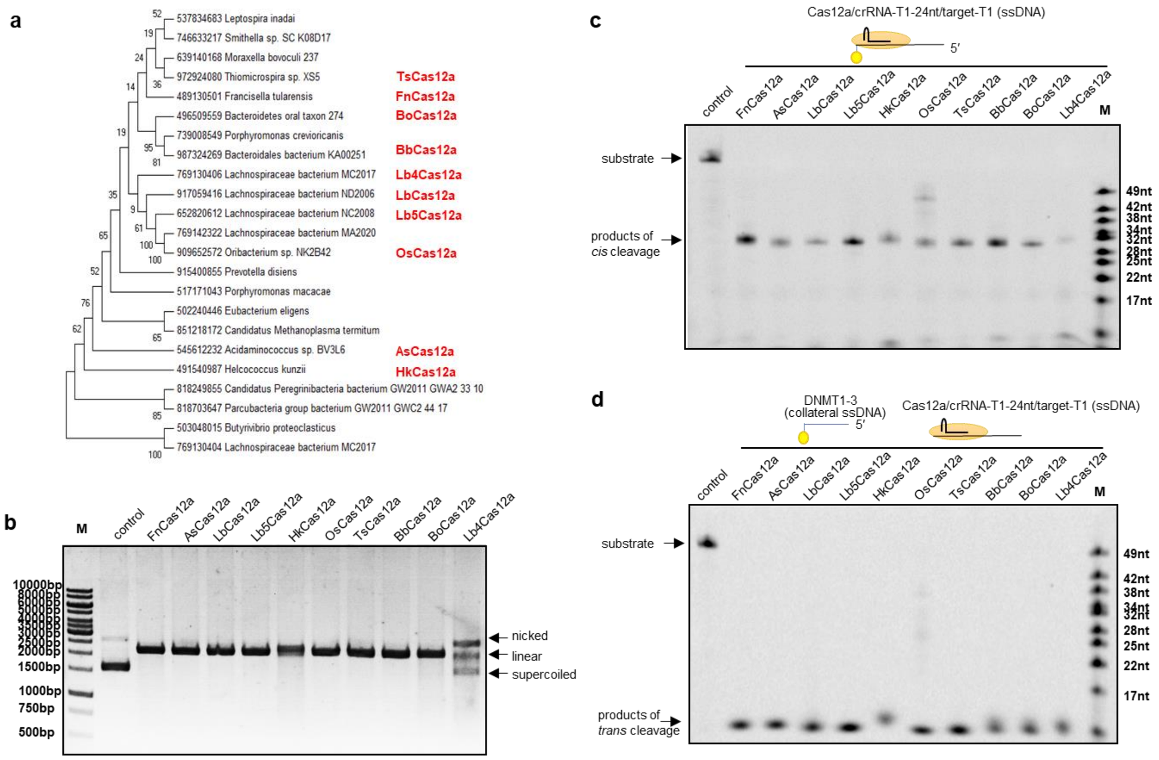

Figure S6 Characterization of the *cis* and *trans* cleavage activities of ten Cas12a proteins from different species. **(a)** A phylogenetic tree of partial Cas12a-family proteins, among which ten were chosen for further biochemical analyses and were indicated in red. **(b)** Cleavage of plasmid pCB1A2_2 by ten different Cas12a proteins with crRNA-T1-24nt. **(c)** *Cis* cleavage of the ssDNA target-T1 by ten different Cas12a proteins with crRNA-T1-24nt. All Cas12a proteins showed *cis* cleavage activity. **(d)** *Trans* cleavage of the ssDNA target-DNMT1-3 by the complex of Cas12a/crRNA-T1-24nt/target-T1 (ssDNA). All Cas12a complexes showed *trans* cleavage activity.

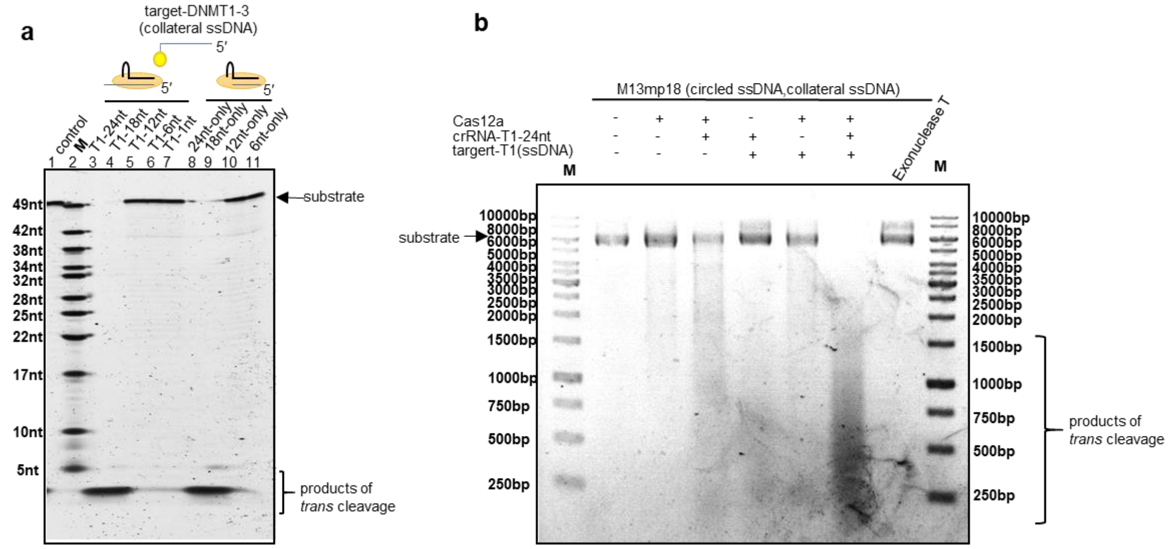

Figure S7 The Cas12a *trans* cleavage activity on ssDNA. **(a)** *Trans* cleavage of collateral ssDNA (target-DNMT1-3) by the Cas12a complex with different lengths of target ssDNA. In lanes 3-7, 3′-extended sequences were designed on the target ssDNA, which was indicated in the schematic; lanes 8-11, there were no extended sequences beyond the 3′ terminal of the target ssDNA. The crRNA-T1-24nt was used in the assay, and detailed information of the target ssDNAs used could be found in Extended Data Table 1. **(b)** Cas12a *trans* cleavage of circular target ssDNA of M13mp18**.** Once the ternary complex of Cas12a/crRNA/target DNA formed, circular ssDNA M13mp18 was promiscuously cleaved. Exonuclease T (Exo T), which is a single-stranded RNA- or DNA-specific nuclease that requires a free 3′ terminus and removes nucleotides in the 3′ to 5′ direction, showed no cleavage activity on circular M13mp18 and was therefore employed as a negative control.

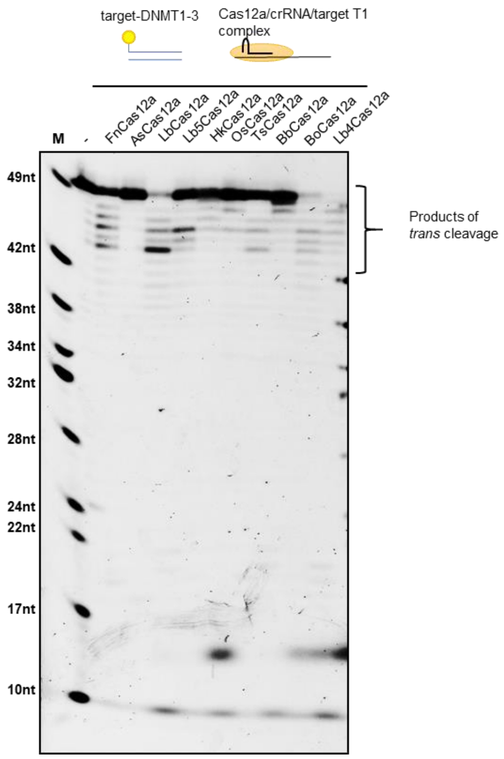

Figure S8 *Trans* cleavage of dsDNA substrate by ten different Cas12a complexes. Upon the formation of Cas12a/crRNA-T1/target-T1 (ssDNA), most Cas12a complexes (except AsCas12a) had *trans* cleavage activity on the ends of dsDNA substrate of target-DNMT1-3, among which the activity of LbCas12a, BoCas12a and Lb4Cas12a complexes was much higher.

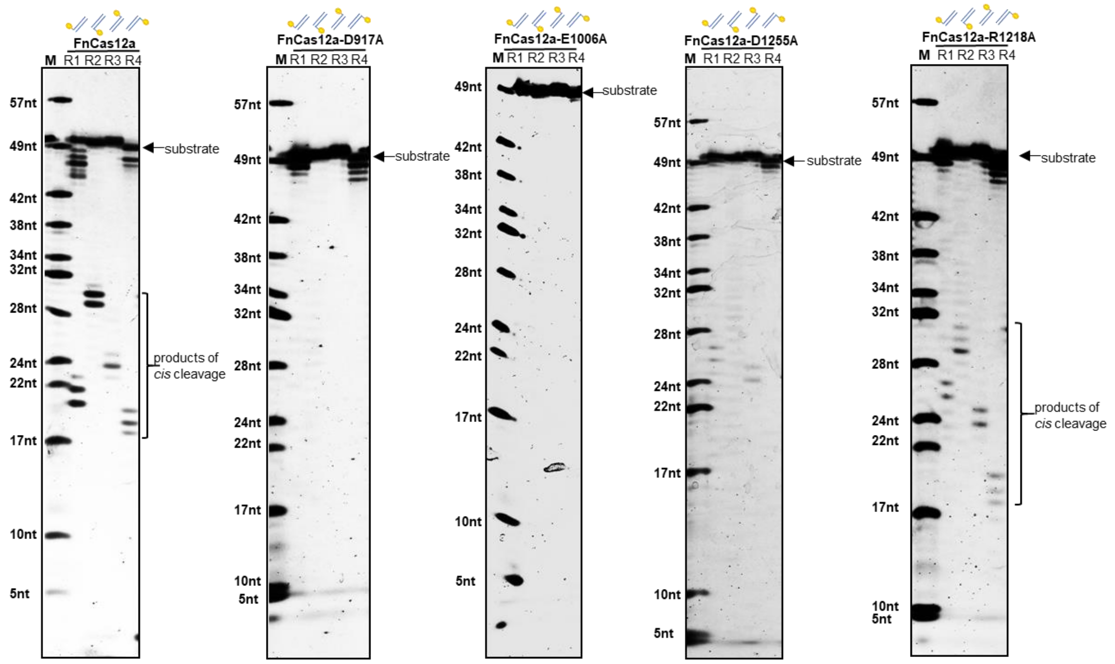

Figure S9 Identification of the cleavage sites in double-stranded DNMT1-3 by FnCas12a and its mutants (D917A, E1006A and D1255A mutations in the RuvC domain; R1218A mutation in the Nuc domain), employing crRNA-DNMT-23nt and FAM-labelled dsDNA. Labelling of targets was indicated above each lane, i.e. R1: 5′-labelled non-target strand, R2: 3′-labelled target strand, R3: 3′-labelled non-target strand, and R4: 5′-labelled target strand. All mutants showed either weakened or completely lost activity on dsDNA cleavage.

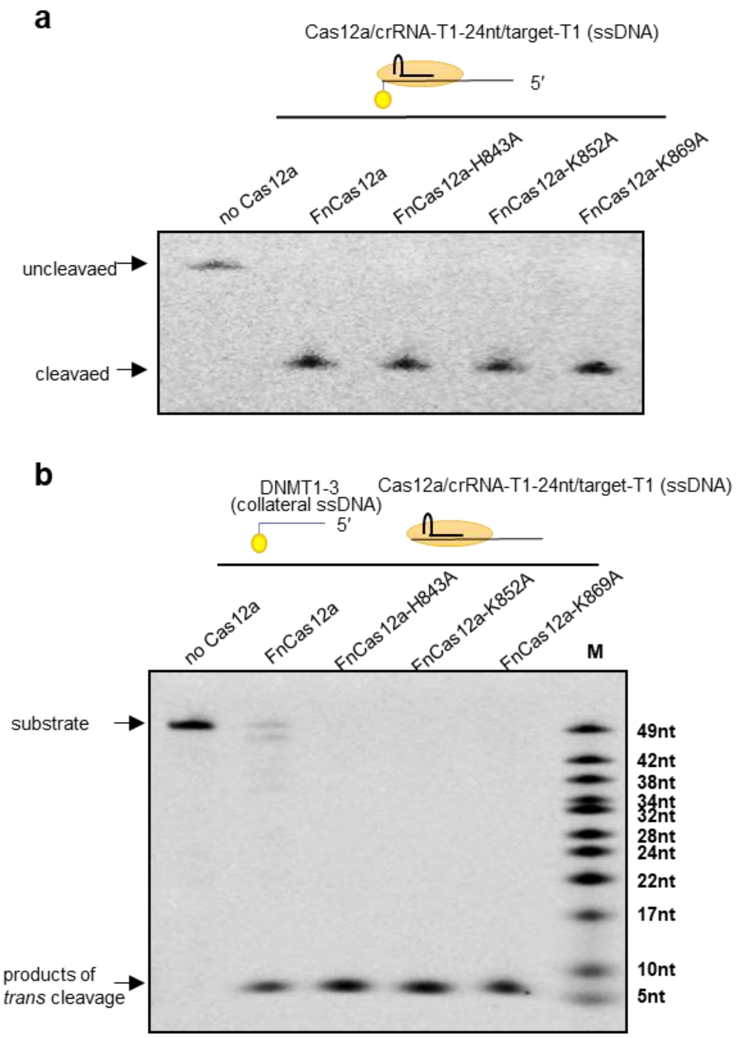

Figure S10 Identification of key residues in Cas12a that were involved in the *cis* and *trans* ssDNA cleavage. **(a)** *cis* cleavage of target ssDNA (target-T1-R-FAM) by FnCas12a and three FnCas12a mutants with single amino acid replacement in H843, K852 and K869, which are associated with the RNase activity. No significant difference was found in the *cis* cleavage activity among the tested Cas12a proteins. **(b)** *Trans* cleavage of the 3′-FAM-labelled collateral ssDNA of target-DNMT1-3-R by the ternary complexes of wild-type FnCas12a or its mutants. The three tested residues (H843, K852 and K869 in FnCas12a) are associated with the RNase activity. No significant difference was found in the *trans* cleavage activities among the tested Cas12a proteins.

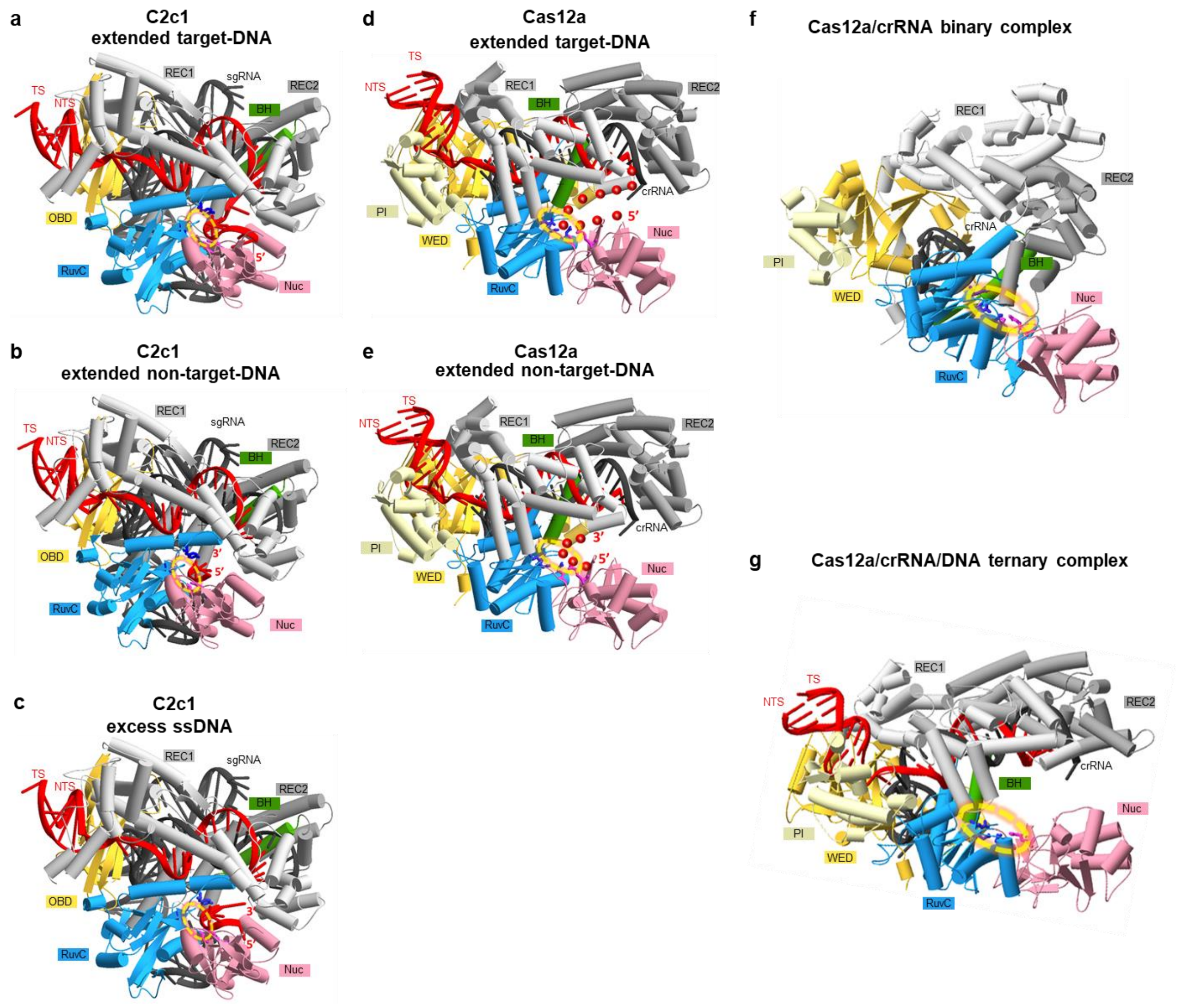

Figure S11 The RuvC catalytic pocket model for C2c1 and Cas12a. **(a-c)** C2c1 complex: the ternary complex C2c1 with sgRNA and extended target DNA (PDB: 5U30 in Figure S11a), extended non-target DNA (PDB: 5U33 in Figure S11b) and excess ssDNA (PDB: 5U31 in Figure S11c). All above substrate DNAs were positioned in the RuvC catalytic pocket (labelled in dashed yellow circle). **(d-e)** Cas12a complex (PDB: 5B43): the ternary complex of Cas12a with crRNA and proposed extended target DNA (Figure S11d) and extended non-target DNA (Figure S11e). Red dots represented the proposed positions of extended target-DNA and non-target-DNA. **(f-g)** Figure S11f represented the binary complex Cas12a/crRNA with a triangle-shaped structure (PDB: 1WJX) and Figure S11g represented the ternary complex Cas12a/crRNA/DNA with a bilobed architecture (PDB: 5B43). Molecular graphic images were prepared using CueMol (http://www.cuemol.org). DNA was colored in Red, RNA was colored in black and the RuvC catalytic pocket was indicated by dashed yellow circles.

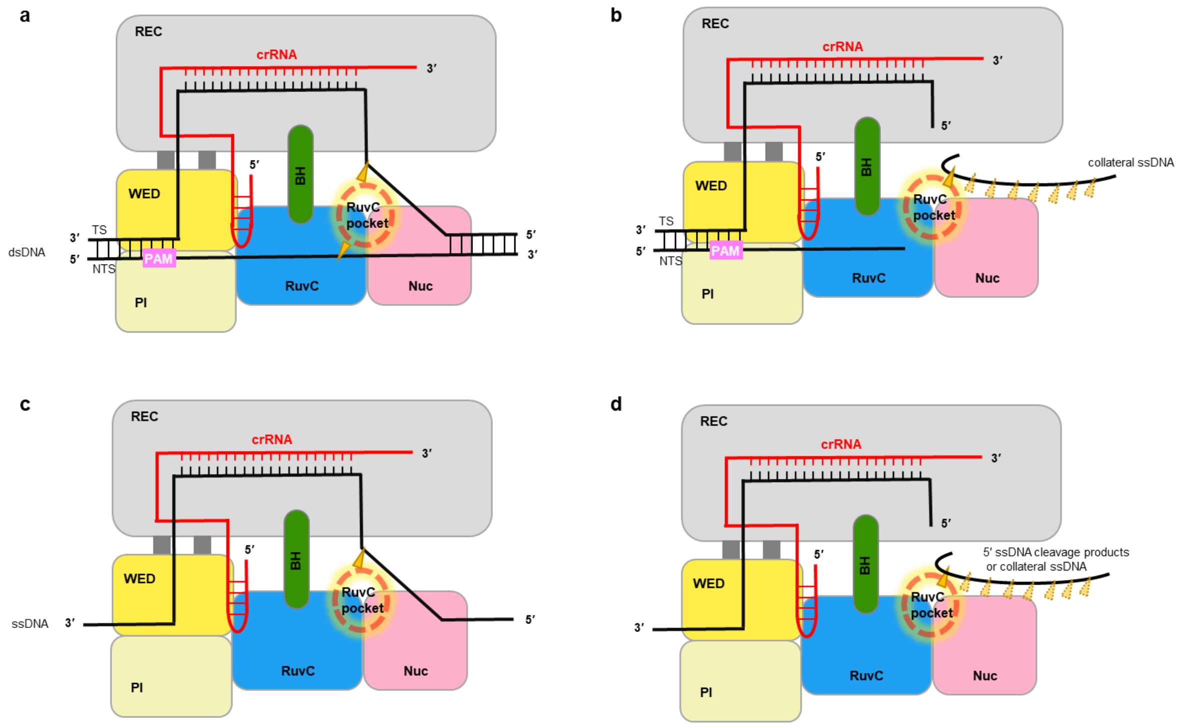

Figure S12 The Cas12a cleavage models. Substrates included target dsDNA **(a)**, collateral ssDNA *trans*-cleaved by the ternery complex of Cas12a/crRNA/target dsDNA **(b)**, target ssDNA *cis*-cleaved by the Cas12a complex **(c)** and *cis*-cleaved ssDNA which was then *trans*-cleaved by the Cas12a complex **(d)**. All substrate DNAs were proposed to be cleaved by the active sites in the Cas12a RuvC pocket.

References

1. Li, S. Y., Zhao, G. P. & Wang, J. C-Brick: A New Standard for Assembly of Biological Parts Using Cpf1. *ACS Synth. Biol.* **5**, 1383-1388 (2016).
2. Lei, C. *et al*. The CCTL (Cpf1-assisted Cutting and Taq DNA ligase-assisted Ligation) method for efficient editing of large DNA constructs *in vitro*. *Nucleic Acids Res.* **45**, e74 (2017).
3. Carneiro, F. R. *et al*. Spectroscopic characterization of the tumor antigen NY-REN-21 and identification of heterodimer formation with SCAND1. *Biochemical and biophysical research communications* **343**, 260-268 (2006).
